# Recent advances in metal-catalysed oxidation reactions

**DOI:** 10.1098/rsos.241215

**Published:** 2025-01-08

**Authors:** Aleena Raju, Subhiksha Jothish, Kokila Sakthivel, Shachi Mishra, R. J. Gana, Kotaro Kikushima, Toshifumi Dohi, Fateh V. Singh

**Affiliations:** ^1^ Department of Chemistry, SAS, Vellore Institute of Technology, Chennai, Tamil Nadu 600127, India; ^2^ P. G. Department of Chemistry, Jai Prakash University, Chapra, Saran, Bihar 841302, India; ^3^ College of Pharmaceutical Sciences, Ritsumeikan University, Kusatsu, Shiga 525-8577, Japan

**Keywords:** metal, catalysis, oxidation, oxidants, environment friendly

## Abstract

Oxidation reactions are vital tools in synthetic organic chemistry. Oxidation of organic species such as alcohols, phenols, aldehydes and ketones provides synthetically valuable organic compounds, especially synthetic intermediates for several biologically active compounds. Some of these synthetic intermediates have shown their synthetic utility in the total synthesis of natural products. Several classical and modern synthetic approaches have been used to achieve these oxidation reactions. In this review article, various oxidation reactions achieved by metal catalysis are highlighted.

## 1. Introduction

Oxidation of organic molecules is a fundamental transformation in organic chemistry, which allows the introduction of specific functional groups within molecules and the formation of complex molecular frameworks, enabling a diverse array of synthetic pathways [1–3]. Representative examples of oxidation reactions include the introduction of oxygen and nitrogen functional groups into hydrocarbons, oxidation of alcohols into carbonyl compounds, oxidation of amines and sulfur compounds, and dehydrogenative bond formations [4–8]. These oxidation processes play a pivotal role in a wide range of chemical industries, including the manufacturing of both bulk and fine chemicals, which are employed to produce raw materials, synthetic intermediates and end products, such as functional materials and pharmaceutical compounds [9–11]. Therefore, efficient methods for oxidative transformations would facilitate the design and development of novel functional organic molecules. In this context, the remarkable attributes of transition metal catalysts have garnered significant interest as they provide innovative approaches to improve reaction yields, selectivity and environmental impact [12–15]. This review article will focus on the recent advances in transition metal-catalysed oxidation reactions of organic compounds. In this systematic review, we have included reports published from 2016 onwards. The pursuit of these oxidation methods undoubtedly contributes to the development of sustainable synthetic methodologies and the advancement of the chemical sciences.

## 2. Nickel-catalysed

Nickel complexes are well known for their catalytic activity. Recently, novel metal–organic framework (MOF)-based and nanomaterial-based nickel catalysts have shown improved efficiency as well as recyclability [16,17]. Zhou *et al.* used a nickel-based catalyst in the presence of an oxidant, *tert*-butyl hydroperoxide (TBHP), to aid the oxidation of secondary alkanes to ketones. The nickel nanoparticles embedded in nitrogen-doped carbon material-based catalyst were prepared via thermolysis of a Ni-containing MOF under inert atmosphere. The catalyst was found to be selective even in the presence of electron-withdrawing groups (EWGs) and electron-donating groups (EDGs). Furthermore, it could be recycled and reused four times without losing any activity [18].

**Scheme 1.**
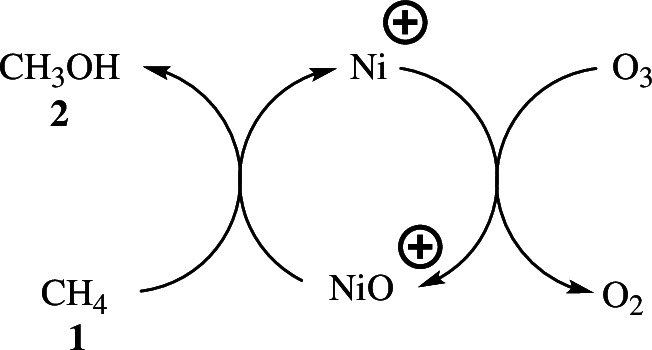
The nickel cation-mediated oxidation of methane **1** to methanol **2**.

For the oxidation of methane to methanol, McDonald *et al*. produced a cationic nickel catalyst. The oxidation followed a catalytic cyclic pathway, with the initial step being the oxidation of Ni^+^ to NiO^+^ by ozone, followed by the oxidation of methane **1** to methanol **2** with Ni^+^ regeneration (Scheme 1) [19].

The oxidation of alcohols is one of the most important applications of nickel catalysts. An inert nickel foam (NF) was changed to NiO/NF as a catalytically active monolith electrode by simple ultrasonic acid treatment and air oxidation at room temperature, leading to a uniform NiO nanosheet array generated on the surface of NiO/NF. The active NiOOH species is reduced to Ni(OH)_2_ by simultaneously oxidizing methanol to high-value-added products such as formaldehyde, carbonate and formic acid once the methanol molecules adsorb onto the electrocatalyst surface. Through an Eley–Rideal-like process, this catalyst also promotes the oxidation of carbon monoxide to carbon dioxide [20].

For the oxidation of urea, hollow sodium nickel fluoride nanocubes coated on multiwall carbon nanotubes (SNF–MWCNT) generated by a simple microwave (MW)-assisted technique using an *in situ* template mechanism were used. Because the electro-catalytic surface area of the catalysts was proportionate to the charge required to reduce Ni^3+^ to Ni^2+^, the process was comparable to that of the Ni(OH)_2_ electrode following the reaction mechanism:


$$6\;Ni^{2+}\;(s)+18\;OH^{–}(aq)\to 6\;NiOOH(s)+6\;H_{2}O(l)+6\;e^{–},$$

$$6\;NiOOH(s)+CO(NH_{2}{)}_{2}(aq)+2\;OH^{–}(aq)\to 6\;Ni(OH{)}_{2}(s)+CO_{3}^{2–}(aq)+N_{2}(g),$$

$$CO(NH_{2}{)}_{2}(aq)+8\;OH^{–}(aq)\to 6\;H_{2}O(l)+CO_{3}^{2–}(aq)+N_{2}(g)+6\;e^{-}.$$

The catalyst has strong catalytic activity for urea oxidation because the Ni^3+^ species drive the generation of NiOOH. Furthermore, the SNF has been proposed as a possible anode catalyst for direct urea oxidation fuel cells, making the catalyst a viable contender [21].

Another nickel-assisted urea oxidation was carried out by using a two-dimensional (2D) MOF including nickel species and an organic ligand of benzenedicarboxylic acid, similar to the previous process. In this framework, the nickel cations have a high oxidation state, which produces strong interactions between Ni species and benzenedicarboxylic acid ligands, making it a possible electrocatalyst for urea oxidation at the electrode surface, resulting in carbon dioxide and nitrogen bubbles [22].

## 3. Ruthenium-catalysed

**Scheme 2.**
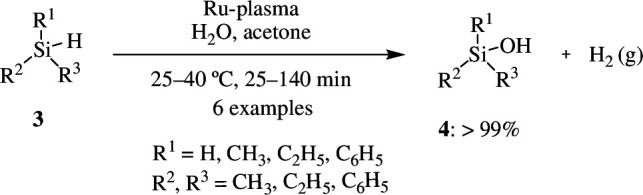
Oxidation of organosilane **3** to silanol **4** with water by using supported Ru catalyst.

Ruthenium is a cheaper transition metal and thus gives rise to less expensive catalysts, displaying competitive catalytic efficiency. Gnanakumar *et al*. used plasma-assisted synthesis to create an ultrafine ruthenium nanocatalyst (Ru-plasma) with monodispersed Ru nanoparticles loaded onto carbon and TiO_2_ supports. The catalyst facilitates the oxidation of organosilane **3** to organosilanol **4** with 99% selectivity, using water as the principal oxidant at room temperature (Scheme 2). The catalyst outperforms traditional methods because it does not produce disiloxanes through silanol condensation and can be utilized for oxygen evolution reactions [23].

Apart from organosilane oxidation, a homogeneous ruthenium phosphorus-based catalyst (RuH(CO)Cl(PPh_3_)_3_) is employed for the selective oxidation of alcohol. With molecular oxygen as the principal oxidant and no base or hydrogen/electron transfer agents, this catalyst aids in the smooth oxidation of secondary benzylic, allylic, heterocyclic and aliphatic alcohols. It was also reported that even for N-, O- and S-substituted heterocycles and aryls with EDGs and EWGs, the oxidation was successful. However, the reaction is less effective for NH_2_-substituted benzyl alcohol substrates and primary aliphatic alcohols, as the former produce no product owing to chelation, while the latter produces homo-coupled esters rather than aldehydes. The reactivity of the alcohol oxidation follows the order: 1° benzylic > 2° benzylic > 1° aliphatic > 2° aliphatic. Thus, the catalyst is shown to be efficient since it oxidizes 1° and 2° alcohols to their corresponding aldehydes and ketones with 100% selectivity, low catalyst loading and inexpensive ligands, while avoiding the formation of by-products by over-oxidation [24].

Ru_3_/CN is another ruthenium-based catalyst for alcohol oxidation with good conversion and chemoselectivity. It was made in a two-step process, with the first step involving encapsulating and separating pre-selected metal cluster precursors using molecular-scale zeolite imidazole frameworks, and the second step, pyrolysis to remove ligands from metal cluster precursors and forming nitrogen-doped porous carbon to stabilize the clusters. Even in the presence of EWGs, EDGs and halogens, the catalyst preferentially oxidizes 2-aminobenzyl alcohol to 2-aminobenzaldehyde. When the reaction is carried out for a longer time, it also gives corresponding aldehydes with a yield of 93–94% for allylic alcohols and N-heterocyclic benzyl alcohols. Furthermore, the catalyst outperforms previous ruthenium-based catalysts such as Ru_1_/CN, Ru NP/CN and Ru/C because the uniform Ru_3_ clusters operate as the main active sites for the oxidation reaction [25].

**Scheme 3.**
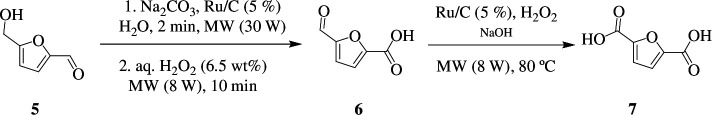
Ruthenium catalysed microwave-assisted oxidation of 5-hydroxymethylfurfural **5** to 2,5-furandicarboxylic acid **7** via 5-formyl-2-furancarboxylic acid **6**.

Recently, Zhao *et al*. discovered that 5% Ru/C can aid in the formation of 2,5-furandicarboxylic acid (FDCA) **7**, a terephthalic acid alternative used in the manufacture of polyethylene terephthalate. Three distinct routes (Scheme 3) are involved in the oxidation producing FDCA from 5-hydroxymethylfurfural (HMF) **5**. In the presence of Na_2_CO_3_ as a base and H_2_O_2_ as an oxidant, the Ru/C catalyses the oxidation of the primary hydroxyl group of HMF to the corresponding aldehyde to generate 2,5-furandicarboxaldehyde. Further, 5-formyl-2-furancarboxylic acid (FFCA) **6** is formed when one of the formyl groups is changed to a carboxyl group. The presence of a base promotes the oxidation of aldehyde groups in HMF via nucleophilic OH^−^ attack of groups, resulting in significant FFCA **6** yields even at atmospheric pressure and in short reaction durations. Despite this, because the HMF is rapidly decomposed to its side products and Na_2_CO_3_ is insufficient to oxidize the FFCA **6** to FDCA **7**, NaOH is added as the reaction’s base. Under MW conditions, the Ru/C oxidizes HMF to FFCA in 10 min as a result of nucleophilic attack of the OH^−^ group of NaOH at low pressure. As a result, the researchers demonstrated that the same catalyst may successfully oxidize the reactant by varying the kind and amount of base [26].

## 4. Manganese-catalysed

**Scheme 4.**
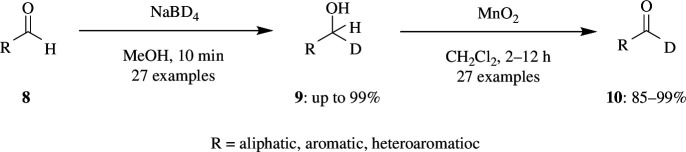
Selective oxidation of alcohol-d_1_ **9** to aldehyde-d_1_ **10** using MnO_2_.

Selective oxidation of alcohol-d_1_ **9** to aldehyde-d_1_ **10** by means of NaBD_4_ reduction followed by MnO_2_ oxidation has been developed by Okamura *et al*. The range of aldehyde-d_1_ derivatives includes aromatic and unsaturated aldehydes. The NaBD_4_/MnO_2_ was found to be very effective and selective, oxidizing alcohols with other functional groups like halide, ether, acetal, nitrile, nitro, esters and alkyne groups on the aromatic ring with 98% yield (Scheme 4). Even acrolein and propynal were reduced and oxidized in a smooth manner [27].

The NaBD_4_ is used as D^−^ source in preference to LiAlD_4_, as the latter is reactive and reduces other functional groups like nitrile, ester, nitro, acid moieties and alkene and alkyne with EWGs. Moreover, a low temperature of −20°C is required for the LiAlD_4_ reduction to avoid the undesired 1,4-reduction. The NaBD_4_/MnO_2_-based oxidation is the best reagent so far reported for the oxidation of phenylbenzylalcohol. Within the different alcohols, the oxidation of propargyl alcohols results in a higher percentage of D compared with others. This is because the propargyl alcohol C–H cleavage is slower than other alcohols, according to the ^13^C kinetic isotope effect. Thus, the catalytic oxidation of these alcohols finds application in various organic reactions when used as synthetic intermediates.

Lu *et al.* developed a graphene–MnO_2_ (G–Mn) catalyst that can oxidize formaldehyde at various temperatures. The catalyst was created by self-assembling MnO_2_ onto the surface of graphene nanosheets, which helped expose more MnO_2_ surfaces for reagent adsorption and desorption, as well as providing more active sites for catalysis. The G–Mn has the best catalytic activity at 65°C, but conversion was 100% at 70, 90 and 140°C. The graphene’s 2D structure and hydroxyl groups make it easier for formaldehyde and O_2_ molecules to bind to the hybrid surface, where the formaldehyde is oxidized to generate formate intermediate while oxygen activation and oxygen transfer through redox cycles between manganese species (Mn^4+^/Mn^3+^) take place. The formate is then directly oxidized to CO_2_ along with a water molecule by the surface hydroxyl groups of graphene under various circumstances, as illustrated in the following equation:


$$HCOO\;-\;Mn+Mn\;-\;OH\to H_{2}O+CO_{2}+2Mn.$$

As a result, graphene acts as a support, introduces high dispersion of MnO_2_ nanoparticles and provides a large number of active centres for reactants, resulting in high efficiency and catalytic performance of the supported catalyst. Furthermore, even after five cycles of on/off, the catalyst showed no catalytic loss and thus had resisted deactivation [28].

Sun *et al*. developed four Mn-based catalysts for toluene oxidation in the presence of oxygen. MnOx–CeO_2_–MOF and MnOx–MOF catalysts are made from MOF produced by *in situ* pyrolysis of Mn–MOF-74 precursor, while MnOx–CeO_2_–CP and MnOx–D catalysts are made by MnOOH co-precipitation and thermal decomposition, respectively. MnOx–CeO_2_–MOF > MnOx–MOF > MnOx–CeO_2_–CP > MnOx–D is the order of catalytic activity. The addition of Ce to MnOx resulted in a high surface area, a large number of oxygen vacancies, good low-temperature reducibility and great oxygen mobility, all of which contributed to the increased activity of MnOx–CeO_2_–MOF. Additionally, the high surface Mn^4+^ concentration and diffusion, as well as the dispersion of cerium ions, which resulted from the unique pore geometries and intrinsic surface features of Mn–MOF-74, contribute to the catalytic activity of toluene oxidation. The catalyst follows the Mars–van Krevelen mechanism for toluene oxidation, which involves the interaction of adsorbed toluene with lattice oxygen to form CO_2_ and H_2_O as reaction products, as well as the replacement of generated oxygen vacancies by O_2_ [29].

**Scheme 5.**
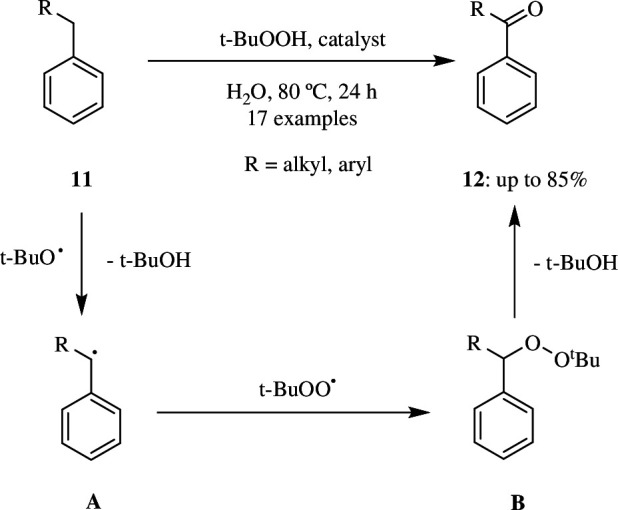
Oxidation of alkylbenzene **11** with *tert*-butyl hydroperoxide over manganese-based metalloporphyrin-based porous coordination polymer (Mn^II^(TCP)Mn^II^) catalyst.

Apart from MnO_2_-based oxidants, the manganese-based metalloporphyrin-based porous coordination polymer (Mn^II^(TCP)Mn^II^), where TCP is 5,10,15,20-tetra(4-(phenoxy-4-yl)butanoic acid), can also be used as a suitable catalyst for the oxidation of alkylbenzene **11** to ketone **12**. In the presence of TBHP as an oxidant, the catalyst is shown to be selective and yields 99% of the product. The catalyst acts as an initiator for the homolysis of TBHP into free alkoxy and alkylperoxy radicals. Following that, alkylbenzene **11** undergoes oxidation at the α-position, resulting in ketones **12** (Scheme 5). The catalyst can be filtered and reused three times, but there is a slight drop in catalytic activity after the third use, with no apparent catalyst leaching [30].

## 5. Rhodium-catalysed

**Scheme 6.**
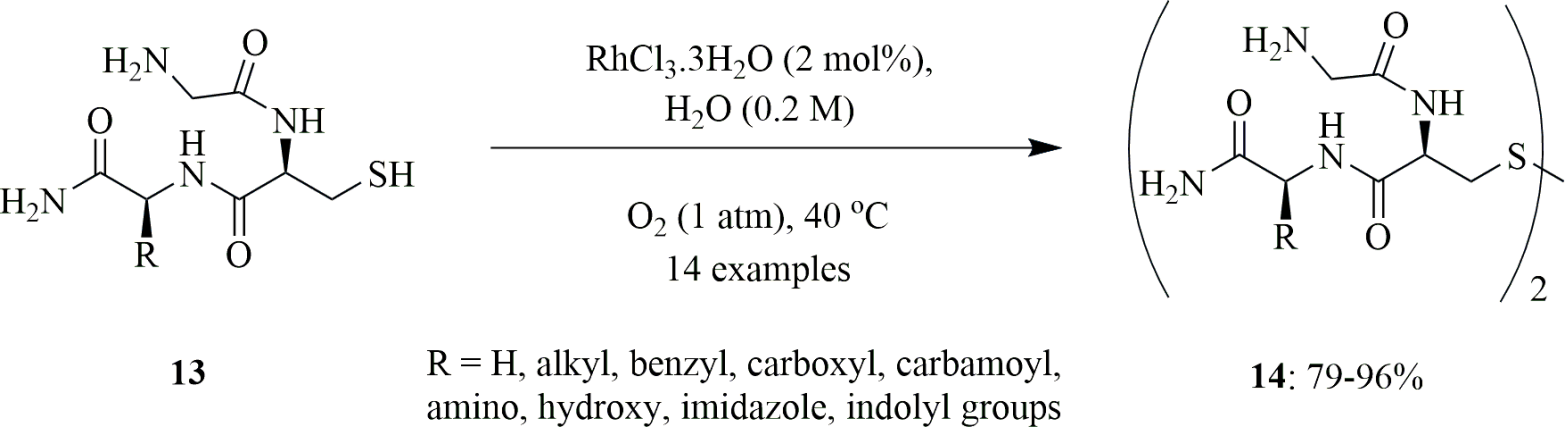
Rhodium-catalysed oxidation of unprotected peptide thiols **13** to disulfides **14** with oxygen in water.

**Scheme 7.**
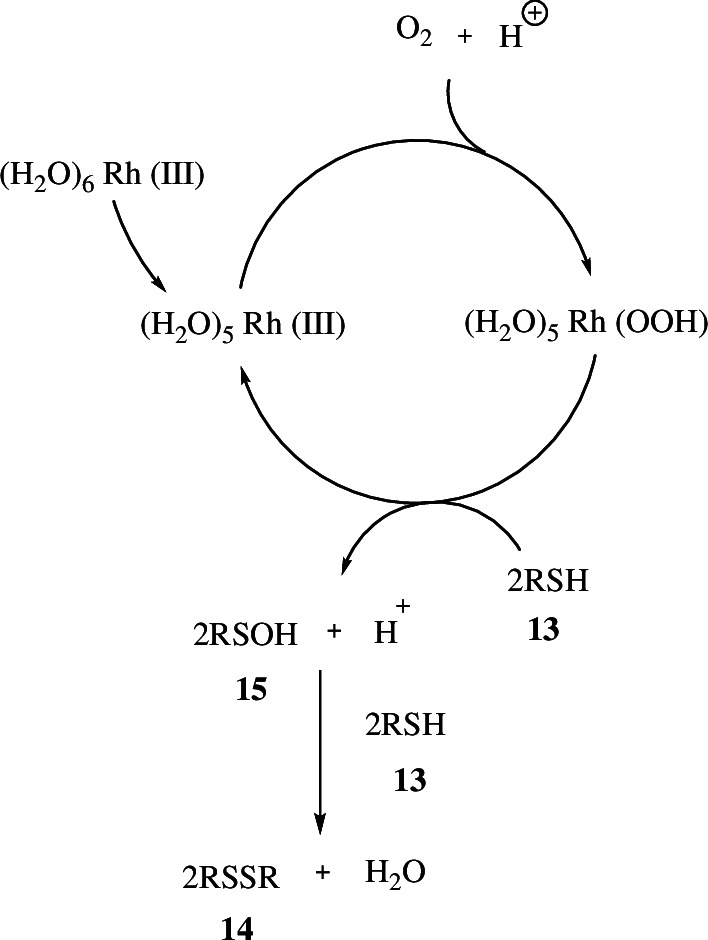
Proposed reaction mechanism of oxidation of **13** to **14**.

Arisawa *et al*. reported the oxidation of peptide thiols **13** to disulfides **14** using rhodium (RhCl_3_·3H_2_O) as catalyst in water under ambient pressure of oxygen (1 atm; Scheme 6). The reaction is found to be effective at low concentration of rhodium catalyst, because of its applicability to less-water-soluble peptides. Moreover, the insoluble nature of the rhodium catalyst gave a 94% yield when the reaction proceeded in polar organic solvents like dimethylsulfoxide (DMSO) and trifluoroacetic acid (TFA). The reaction was found to be very effective towards the oxidation of tripeptides or tetrapeptides into their corresponding disulfides when the former were reacted in water at 40°C for 1.5 h under oxygen (1 atm) in the presence of RhCl_3_·3H_2_O (2 mol%). But when the tripeptides contain phenol and heteroaryl groups, 5 mol% of the catalyst should be used to obtain a significant yield. The reaction is highly selective as it only oxidizes the thiols to their corresponding disulfides, without oxidizing other functional groups like hydroxyl, carbamoyl, carboxyl, amino, alkyl, benzyl, phenoxy, sulfide and imidazolyl. The rhodium-catalysed oxidation was also applied to the hexapeptide Cys−Ser−Try−Gly−Ala−Ser−NH_2_, to give the corresponding disulfide in 87% yield. The proposed mechanism of the reaction is as Scheme 7 [31].

The RhCl_3_ in water provides an equilibrium mixture of Rh(III)Cl_*n*_(H_2_O)_6−*n*_ containing Rh(III)(H_2_O)_6_, which then dissociates to form Rh(III)(H_2_O)_5_ and reacts with oxygen to form hydroperoxorhodium complex Rh(III)(H_2_O)_6_(OOH). The complex then reacts with two molecules of thiol (RSH) **13** to form two molecules of sulfenic acid (RSOH) **15** by regenerating Rh(III)(H_2_O)_5_. The sulfenic acid in return reacts with the thiol to form disulfide (RSSR) **14** and water.

**Scheme 8.**
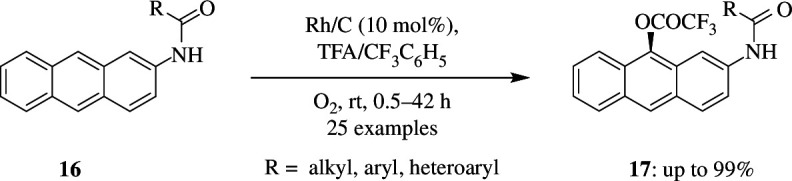
Rh/C-catalysed oxidative trifluoroacetoxylation of anthracene **16**.

Matsumoto *et al*. reported that, using heterogeneous rhodium on carbon catalyst, C–H of anthracenes can be trifluoroacetoxylated and dichloroacetoxylated according to the conditions (Scheme 8). It was reported that the oxidation is regioselective as the trifluoroacetoxylation of anthracene **16** occurs at the 9-position, particularly when TFA is used as solvent. The introduction of electron-withdrawing substituents such as amido groups into the reactants can prevent over-oxidation, resulting in the preferential formation of mono-oxygenated products **17**. Moreover, when a dimethoxy-substituent was introduced on the 2,4 position of the benzene ring of amido groups present at the 2-position of anthracene, a yield of 93% of the final product could be achieved [32].

**Scheme 9.**
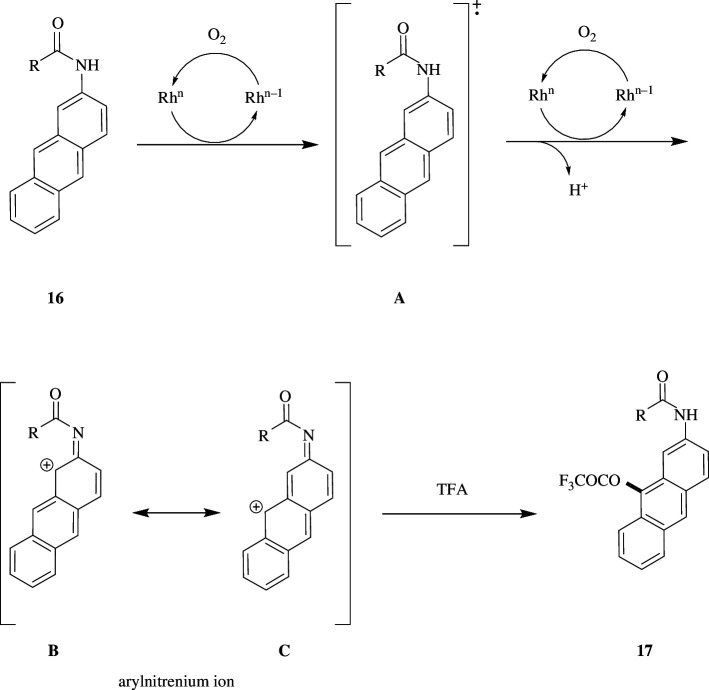
Reaction mechanism of Rh/C-catalysed oxidative trifluoroacetoxylation.

However, electron-donating substituents lower the oxidation potential of aromatic compounds. When 2-*tert*-butylanthracene undergoes trifluoroacetoxylation and dichloroacetoxylation, the regioselectivity is decreased, and it gives rise to a mixture of products at the 9- and 10-positions. Similarly, halogenated anthracenes at the 2-position on oxidation are moderately selective. The proposed mechanism of the reaction is shown in Scheme 9. The amide **16** gets oxidized by the catalyst to produce radical cation intermediate **A**, whereas the reduced catalyst undergoes oxidation by molecular oxygen to regenerate the catalyst. The generated cation radical undergoes successive one-electron transfer and rapidly deprotonates to give rise to arylnitrenium ion intermediate **B**, which then forms its stable resonating structure **C** and reacts with TFA at the 9-position in a selective manner to form the desired product **17**. In contrast, the reaction of anthracene with *tert*-butyl group and halogen at the 2-position involves cation radical intermediates rather than the arylnitrenium ion to exhibit significant regioselectivity.

Apart from the selectivity, the catalyst can be recycled six times without losing its efficiency, being removed from the reaction by centrifugation followed by washing with ethyl acetate. The oxidation is found to be effective towards the trifluoroacetoxylation of pyrene, which is then easily hydrolysed to give hydroxypyrene. Even dicloroacetoxylation of pyrene was also obtained with excellent yield when the TFA was replaced by dichloroacetic acid. Moreover, the reaction is also used for the oxidation of perylene and naphthalene. Thus, the method is more advantageous when compared with the existing C–H bond oxygenation reactions owing to the mild conditions and recyclability of the catalyst.

## 6. Chromium-catalysed

**Scheme 10.**
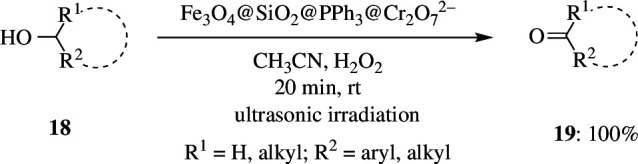
Oxidation reactions of alcohols **18** under ultrasonic irradiation in the presence of$Fe_{3}O_{4}@SiO_{2}@PPh_{3}@Cr_{2}O_{7}^{2-}$nanocomposite.

Maleki *et al*. reported a green, effective and selective nanocatalyst for the rapid oxidation of alcohols **18** such as benzyl alcohol and cyclohexanol to the corresponding aldehyde or ketones **19** (Scheme 10). This method is also found to assist the epoxidation of alkenes. It was founded on fixed on Fe_3_O_4_ magnetic nanoparticles through triphenylphosphine and a silica shell and used in combination with H_2_O_2_ in CH_3_CN under ultrasonic irradiation conditions to produce high yields and quick reaction durations at room temperature. The scheme has several advantages, including mild conditions, fast reaction times, high conversions, easy separation of the nanocatalyst with an external magnet, and reusability of the magnetic composite nanocatalyst for at least five times recycling without considerable loss of catalytic activity [33].

**Scheme 11.**
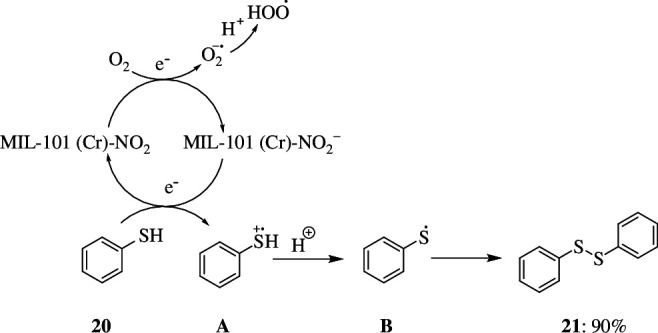
Solvent-free oxidative coupling of thiophenols to disulfides.

MIL-101(Cr)-H MOF was modified in the presence of electron donor (CH_3_ or NH_2_) and withdrawing (NO_2_, Cl or SO_3_H) substituents on the terephthalate organic ligand by Cristina *et al*. It was reported that the MIL-101(Cr)-NO_2_ was the most active catalyst among these as it promotes the solvent-free aerobic oxidation of benzenethiol **20** to diphenylsulfide **21** and the aerobic oxidation of dibenzothiophenes (DBT) **22** in *n*-dodecane or commercial diesel, respectively. Interestingly, for both reactions, no new products were observed. Scheme 11 depicts the chemical route for the solvent-free oxidative coupling of thiophenols **20** to disulfides **21**, in which the electron-rich SH functional transfers an electron to MIL-101(Cr)-NO_2_ and the O_2_ gets reduced to O^2−^, to restore the catalytic cycle. Then, by the intermediacy of the corresponding reactive oxygen species, the O^2−^ can lead to the synthesis of HOO· radicals, which will eventually generate H_2_O_2_ and/or H_2_O by a sequence of electron and proton transfer steps. Meanwhile, the reactive oxygen species would react with thiophenol to generate the phenyl sulfide radical **B**, which would then couple to form disulfide **21**.

**Scheme 12.**
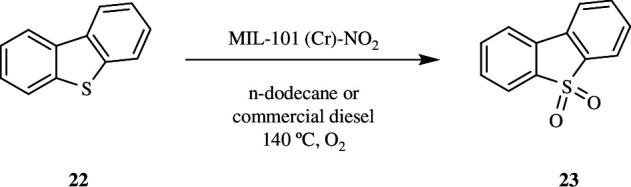
The aerobic oxidation of dibenzothiophene (DBT; **22**) by MIL-101(Cr)-NO_2_ as radical initiator.

Scheme 12 represents the aerobic oxidative desulfurization of fuels utilizing DBT (**22**) as model substrates in *n*-dodecane or commercial diesel as solvent. Thus, the catalytic experiments reveal that the MIL-101(Cr)-NO_2_ acts as a heterogeneous catalyst for the aerobic oxidation of thiophenol (**20**), while acting as a radical initiator for the oxidation of DBT (**22**). Moreover, MIL-101(Cr)-NO_2_ has a higher activity for the reactions than MIL-101(Fe), Cr_2_O_3_ or soluble Cr(III) salts. In all of these circumstances, the catalyst can be reused multiple times without losing its activity, while keeping its crystallinity and undergoing minimal metal leaching [34].

## 7. Molybdenum-catalysed

Owing to the fascinating properties of MOFs, like the tunability of composition and structure to each specific reaction, they are among the most prevalent heterogeneous catalysts. Chen *et al*. created a heterogeneous molybdenum-based MOF catalyst, Mo-NU-1200, in which Mo(VI) is deposited on the Zr node of a mesoporous zirconium-based MOF. When treated with toluene and O_2_ (as oxidant), this catalyst was found to aid in the oxidation of 4-methoxybenzylalcohol to the corresponding aldehyde with 100% selectivity and no acid formation in the absence of a base. However, in the presence of a base, the reaction proceeds, but the conversion drops to 34 ± 4% because the base inhibits the catalytic species reactivity. Furthermore, the catalyst can be regenerated from the solution by washing it with acetone many times and drying it under vacuum without any loss of catalytic activity [35].

**Scheme 13.**
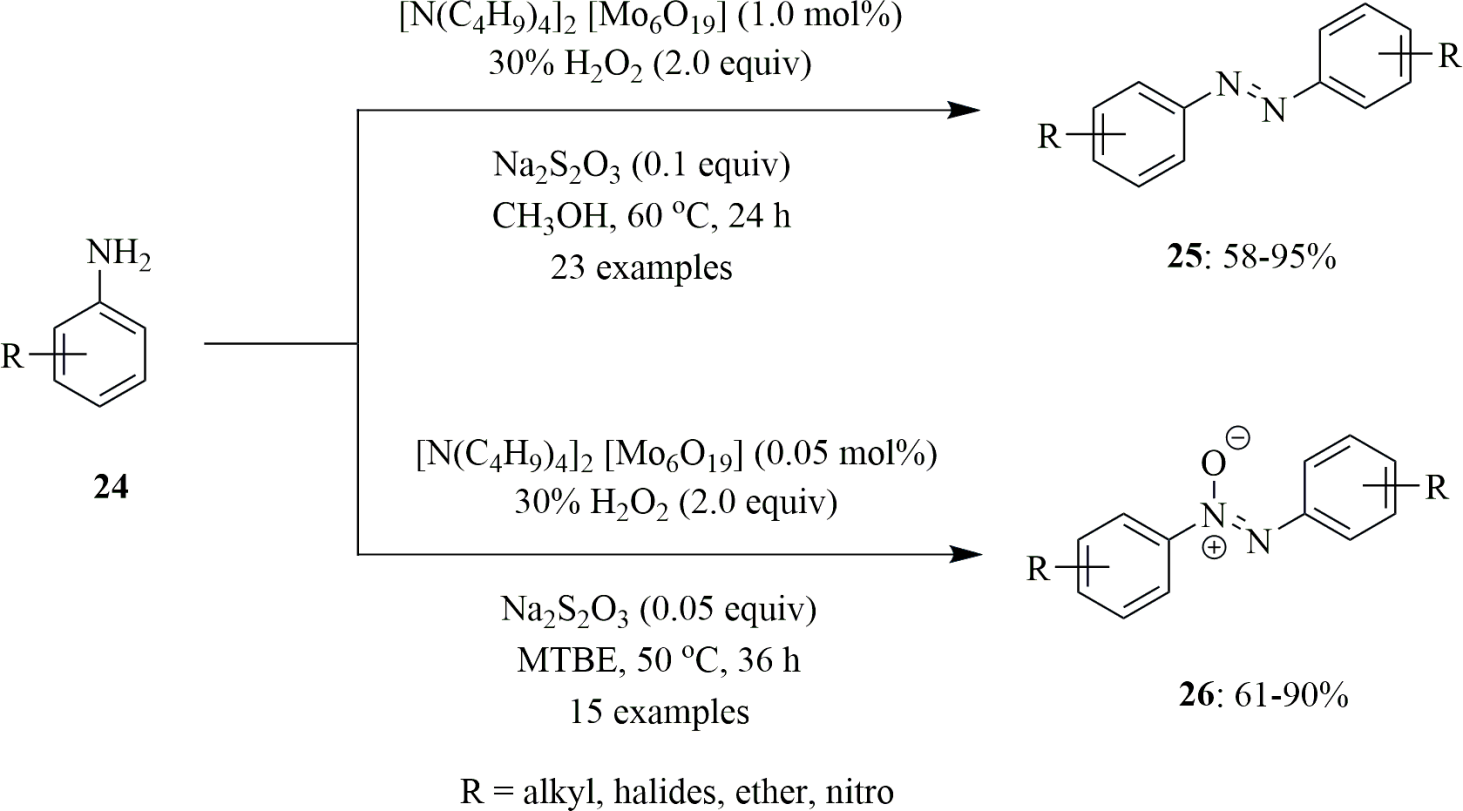
Selective oxidation of aniline **24** to azobenzene **25** and azoxybenzene **26** by Mo oxide catalyst [N(C_4_H_9_)_4_]_2_[Mo_6_O_19_].

By altering the reaction conditions, Han *et al*. produced a Mo oxide catalyst, [N(C_4_H_9_)_4_]_2_[Mo_6_O_19_], for the selective oxidation of aniline **24** to azobenzene **25** and azoxybenzene **26** (Scheme 13). With this catalyst, the oxidation takes place in the presence of the oxidant H_2_O_2_, the additive Na_2_S_2_O_3_ and a suitable solvent. The selectivity in oxidation is due to changes in solvent, catalyst and additive concentration, temperature and reaction time; for example, if the reaction occurs with 1 mol% catalyst in the presence of 0.1 equivalent of Na_2_S_2_O_3_ in methanol at 50°C for 24 h, aniline will undergo oxidative dimerization to give azobenzene **25**; however, if the reaction occurs with 0.5 mol% catalyst in the presence of 0.05 equivalent of Na_2_S_2_O_3_ in methyl-*tert*-butyl ether at 60°C for 36 h, aniline will oxidize to give azoxybenzene **26**. The aniline also undergoes cross-dimerization to yield asymmetric azobenzene **25** with good yield. Since an EWG reduces the electron cloud density of the benzene ring, the yield will be higher for aniline substituted with an EWG compared with an EDG. Also, *para*-substituted products will give better yields than *ortho*- and *meta*-substituted products. The yield of azoxybenzene **26**, on the other hand, declines in the following order: aniline > EDG-substituted > EWG-substituted. Owing to steric effects, the yield of *para*-substituted products is more than those of *ortho*- and meta-substituted compounds, as in the prior situation. Thus, by utilizing the ecologically friendly oxidant H_2_O_2_, the catalyst proves to be selective with excellent yields [36].

## 8. Palladium-catalysed

**Scheme 14.**
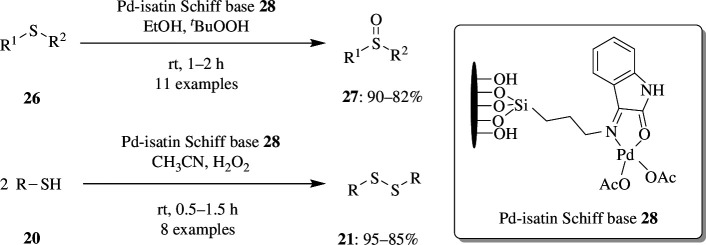
Oxidation of sulfide **26** into sulfoxides **27** and oxidative coupling of thiol **20** to disulfide **21** when exposed to various solvents by heterogeneous palladium-based catalyst **28**.

Oxidation by palladium complexes such as in the Wacker process is one example to have had successful commercial application. These catalysts exhibit excellent selectivity, minimize by-products and maximize yields, and are versatile enough to facilitate various oxidation processes, including those involving alcohols and alkenes. Their recyclability enhances cost-effectiveness, and their ability to operate under milder conditions makes them an environmentally friendly choice. Palladium catalysts can often be recovered from reaction mixtures through methods like filtration or centrifugation, particularly in heterogeneous catalysis. This recovery allows multiple reuses, significantly reducing material costs and waste. Consequently, palladium is not only an economical choice but also a sustainable option in chemical manufacturing, enhancing its appeal in various applications while promoting efficiency and environmental responsibility [37–40]. Recently, Pakvojoud *et al*. created heterogeneous palladium-based catalyst **28** for oxidation of disulfides **26** into sulfoxides **27** and oxidative coupling of thiol **20** to disulfide **21** when exposed to various solvents and oxidants. They immobilized palladium(II)-isatin Schiff base complex within three-dimensional mesoporous silica KIT-6. The reactions give the best yield when the oxidation occurs in ethanol solvent with TBHP as oxidant, whereas the coupling occurs in acetonitrile solvent with hydrogen peroxide oxidant (Scheme 14). The catalyst is found to be chemoselective towards oxidation reaction as it selectively oxidizes sulfides even in the presence of other functional groups and C=C bonds in allyl methyl sulfide and diallyl sulfide. Furthermore, the catalyst **28** may be easily recovered by simple filtration and reused up to five times without losing its effectiveness [41].

Palladium can also catalyse the oxidation of styrene to acetophenone when using a novel cationic palladium(II) compound [(PBO)Pd(NCMe)_2_][OTf]_2_ (where 'PBO' is 2-(pyridine-2-yl)benzoxazole) with 80% yield along with aqueous hydrogen peroxide in ambient air atmosphere. In this reaction, the H_2_O_2_ binds to Pd(II) followed by styrene binding to generate a Pd-alkylperoxide that liberates acetophenone by two competitive processes, one involving a palladium enolate intermediate [42].

## 9. Vanadium-catalysed

Adam reported a vanadium-based catalyst (VOH_2_ID) for the oxidation of alkenes, alcohols and thiophenes. The catalyst is highly effective owing to the high oxidation number of the core V^4+^ ion and the strong affinity of the VO^2+^ ion for the oxygenation reaction. The catalyst when reacted with aqueous hydrogen peroxide (oxidant) and acetonitrile (organic solvent) shows high regioselectivity and chemoselectivity by oxidizing alkene to epoxide, alcohol to aldehyde with acid as minor product, and thiophene to thiophene oxide and thiophene dioxide along with other by-products. The catalyst exhibits catalytic activity even in the absence of a solvent. But in the absence of solvent, water hydrolyses the target product to yield more unknown side products and the oxidant’s water molecules occupy the active coordinated sites of the complex in the catalyst, thereby diminishing the catalytic activity of the VOH_2_ID. Hence, the reaction is favoured in the presence of solvent, with high chemoselectivity [43].

**Scheme 15.**
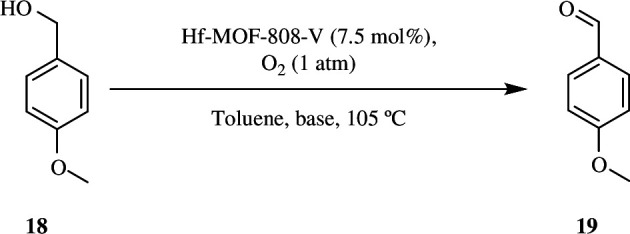
Oxidation of 4-methoxy benzyl alcohol **18** by Hf-MOF-808-V catalyst.

Otake *et al*. synthesized an Hf-MOF-808-V catalyst that oxidizes alcohol **18** to aldehyde **19** with 99% selectivity when treated with O_2_ in toluene at 105°C, where O_2_ is used as oxidant and toluene as solvent. The catalyst was made by integrating vanadium species into Hf-MOF-808, a highly crystalline MOF. The reaction is carried out at 105°C to change the position of a fraction of the V ion in the catalyst framework. The catalyst is also very effective because it may be reused without any catalyst leaching (Scheme 15) [44].

## 10. Iridium-catalysed

**Scheme 16.**
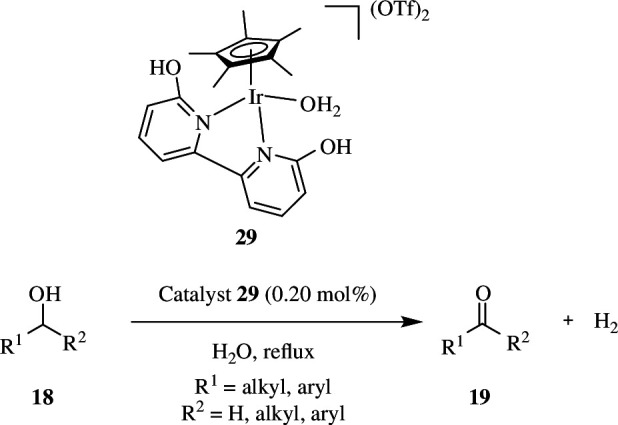
Catalytic conversion of primary and secondary alcohols **18** to corresponding aldehydes and ketones **19**.

For the dehydrogenative oxidation of a range of alcohols, Shimizu *et al.* developed a heterogeneous, reusable iridium-based catalyst. Iridium was immobilized onto a periodic mesoporous organosilica (PMO) catalytic support (BPyOH-BP-PMO) to create the catalyst (Ir@BPyOH-BP-PMO). Because the inside of the PMO pore is relatively hydrophobic owing to the presence of biphenyl groups in the framework, the catalyst outperforms other homogeneous catalysts. As a result, organic substrate approach to the iridium reaction sphere is facilitated, accelerating the dehydrogenative reaction. Primary and secondary alcohols present in aromatic rings, aliphatic groups and sterically hindered substrates **18** were efficiently oxidized to the corresponding aldehydes and ketones **19** with good yields by the catalyst. Furthermore, even when the aromatic rings are substituted with EDGs and EWGs, products with excellent yields can be obtained. The catalyst can also be recovered and reused using simple filtration, with no substantial loss of catalytic activity or selectivity (Scheme 16) [45].

## 11. Photocatalytic reactions

**Scheme 17.**
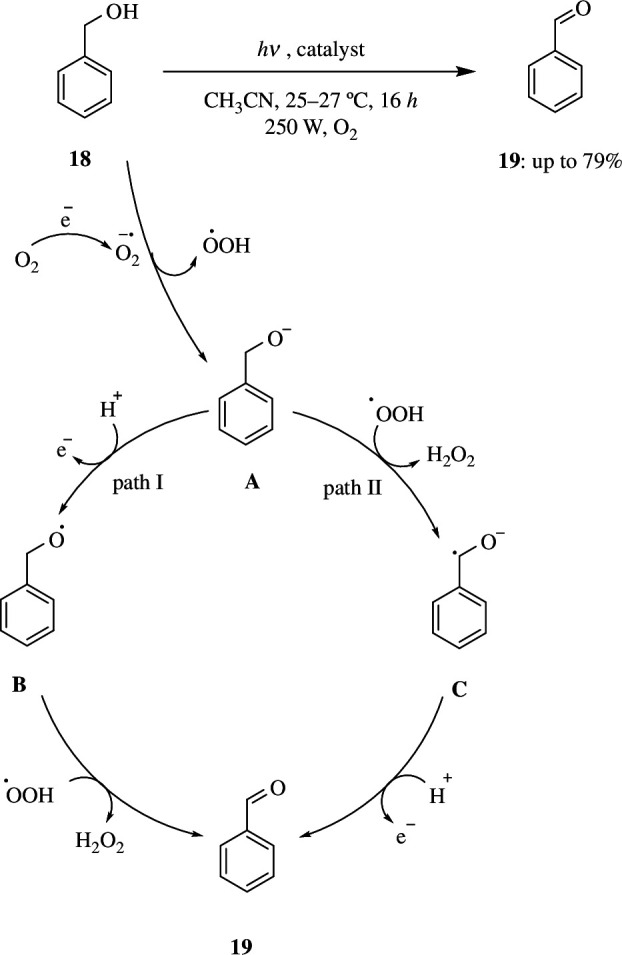
Photocatalytic oxidation of benzyl alcohol over BiVO_4_/g-C_3_N_4_ by an O–H cleavage-mediated, electron-dominated pathway.

For the oxidation of benzyl alcohol, benzylamine and aniline with O_2_, Samanta *et al*. developed a recyclable, effective BiVO_4_/g-C_3_N_4_ catalyst. The g-C_3_N_4_ not only supports BiVO_4_ but also creates an interconnected interface that prevents electron–hole pairs from recombining and provides isolated active sites for catalytic processes. The photocatalytic oxidation of molecular oxygen as an oxidant was reported to be carried out under visible light of wavelength more than 400 nm by altering the composition of BiVO_4_ and g-C_3_N_4_. By following an O–H cleavage-mediated electron-dominated pathway (Scheme 17), the BiVO_4_/g-C_3_N_4_ (5/5) catalyst demonstrates the maximum benzyl alcohol (**18**) conversion with the highest selectivity towards benzaldehyde (**19**).

In this pathway, the electrons generated through light absorption migrate to the conduction band of the catalyst and react with the O_2_ to form superoxide radical anion, which abstracts a proton from benzyl alcohol to form alkoxide anion **A** and HOO^•^. By electron transfer, a positive hole combines with **A** to generate the radical **B**. Later, HOO^•^ interacts with radical **B** to create benzaldehyde (**19**) and H_2_O_2_ (path I). Simultaneously, the alkoxide anion **A** is also expected to react with HOO^•^ to create radical anion **C** and H_2_O_2_, followed by electron transfer; this radical anion **C** can react with the positive hole to generate the product benzaldehyde (**19**) (path II). However, in the presence of methanol, benzaldehyde, benzyl benzoate and methyl benzoate are formed as by-products, with dimethoxymethyl benzene being the main product.

**Scheme 18.**
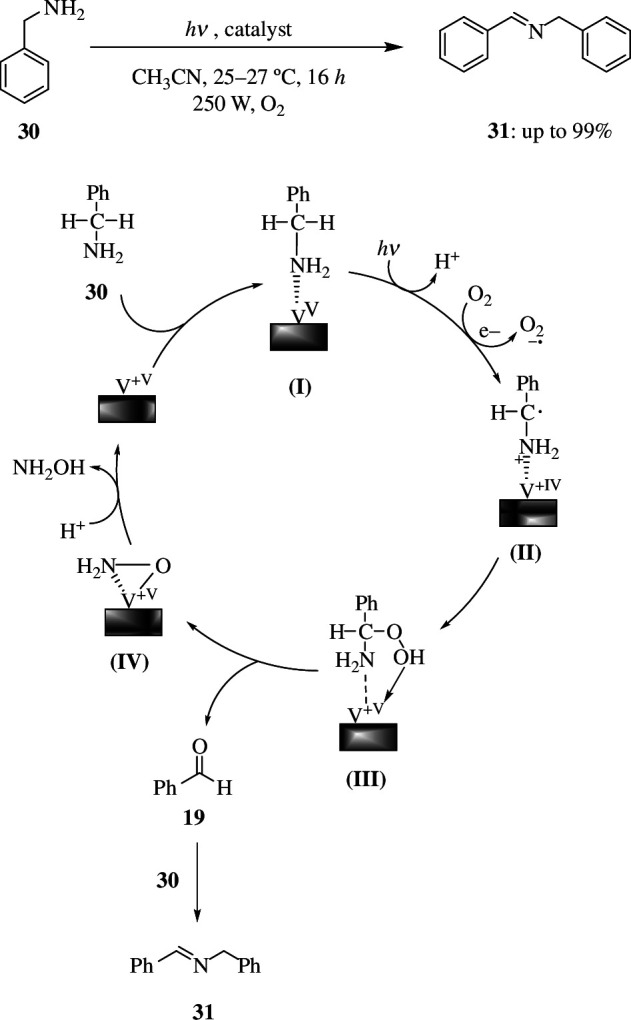
Reaction and mechanism of photocatalytic oxidation of benzylamine (**30**).

The catalyst also converts benzylamine (**30**) to *N*-benzylidene benzylamine (**31**). The mechanism (Scheme 18) involves benzylamine (**30**) adsorption on the catalyst surface, forming a surface complex **(I)**, owing to the electrophilicity of V sites. Upon visible-light absorption, electrons and holes are generated, which get localized on the oxidized substrate, and within the BiVO_4_/g-C_3_N_4_ lattice, respectively. The oxidized substrate is then deprotonated, resulting in the formation of a radical species **(II)**. At the same time, the superoxide radical anion ($O_{2}^{-•}$) is generated by dioxygen and the trapped electron in the conduction band of BiVO_4_/g-C_3_N_4_. Through **(III)**, the **(II)** combines with this $O_{2}^{-•}$ to make benzaldehyde (**19**) and form **31**, which then regenerates the surface V sites of BiVO_4_/g-C_3_N_4_ to complete the photocatalytic oxidation cycle. In the meantime, the aldehyde reacts with benzylamine to form *N*-benzylidene benzylamine, which will be the main product. Even if several functional groups are present, the oxidation yields will not change significantly. However, the yield of electron-rich benzylamines (OMe and Me derivatives) is higher than that of electron-deficient benzylamines (Cl).

**Scheme 19.**
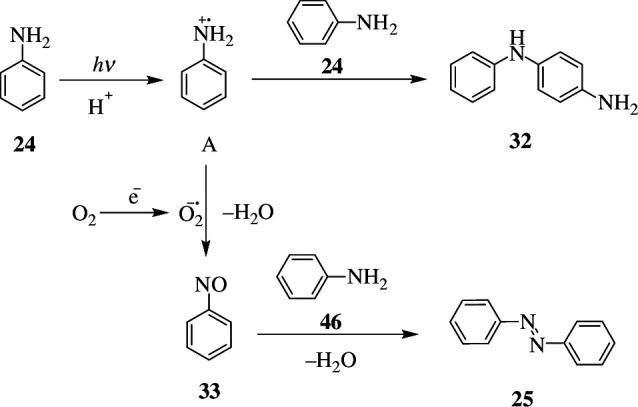
Mechanism of photocatalytic oxidation of aniline (**24**).

Further research showed that photocatalysts convert aniline (**24**) into azobenzene (**25**) and *N*-phenyl benzene-1,4-diamine (**32**) under optimal reaction conditions. Like in usual photocatalysts, electron–hole pairs are generated upon light absorption. The electrons combine with dioxygen to generate superoxide radical anions ($O_{2}^{-•}$), while the holes react with adsorbed aniline molecules to form aniline radical-cation **A** ($PhNH_{2}^{•\text{+}}$). When $PhNH_{2}^{•\text{+}}$ combines with $O_{2}^{-•}$, nitrosobenzene (**33**) is produced, which subsequently condenses with aniline to produce azobenzene (**25**). As a side product, $PhNH_{2}^{•\text{+}}$ (**A**) can react with aniline (**24**) to form *N*-phenyl benzene-1,4-diamine (**32**) (Scheme 19). As a result, the catalyst is viable for the oxidation of benzyl alcohol, benzylamine and aniline, as it is stable and displays little activity loss even after five cycles [46].

Another g-C_3_N_4_-based photocatalyst was prepared by Zhang *et al*., in which Ni-decorated g-C_3_N_4_ hollow spheres (Ni-CNHS) were manufactured using a simple calcination and photoreduction procedure. Under visible-light illumination provided by a 300 W Xenon lamp with a wavelength larger than 420 nm, the catalyst demonstrated outstanding photocatalytic performance and selective amine oxidation. The catalyst converts 61.3% of benzylamine with 99% selectivity towards the synthesis of *N*-benzylidene benzylamine when the optimal loading amount of Ni is 0.41 wt%. It was observed that amines with an EDG on the benzene ring react faster than those with an EWG and that the reaction with sterically encumbered benzylamine with an *ortho*-methyl group yielded the desired product at a slower rate. In contrast to the previously described mechanism, this amine oxidation follows a different pathway, in which photoexcited electrons reduce O_2_ to $O_{2}^{-•}$. Concurrently, photoexcited holes oxidize benzylamine to benzylammonium cation radicals, which combine with oxygen to produce the Ph–CH=NH intermediate and H_2_O_2_. Finally, the Ph–CH=NH intermediate reacts with benzylamine to produce *N*-benzylidenebenzylamine [47].

**Scheme 20.**
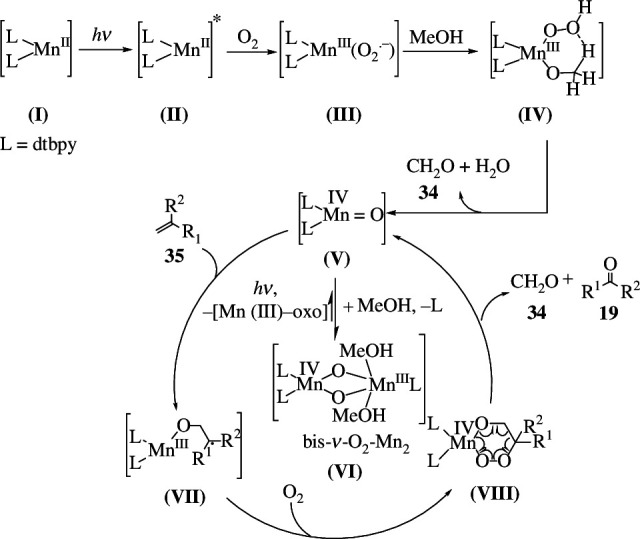
Mechanism for the photo-Mn promoted oxidative cleavage of alkenes **35**.

Under 1 atm of O_2_, a manganese-based photocatalyst, [Mn(dtbpy)_2_(OTf)_2_] (where 'dtbpy' is 4,4′-di-*tert*-butyl-2,2′-dipyridine), was used for selective oxidative cleavage of alkenes to produce ketones. It produces aldehydes and ketones from aromatic and aliphatic alkenes under clean and mild conditions. During the oxidation, halogen, ester, nitro, cyclopropyl, acid, pyridine, boronic ester and alkynyl groups are tolerated. Because it can selectively oxidize dipeptides, urea and glucose-containing alkenes, the catalyst is a promising candidate in bioconjugate chemistry. It was also noted that 3-fluoro-2-methyltridec-1-ene, with a carbon double bond deactivated by an adjacent electron-withdrawing fluorine group, could not offer cleavage as the catalyst is sensitive to substrate electrical effect. Furthermore, the benzylic and tertiary C–H bonds in the substrates remained intact during oxidation, implying that the catalyst is chemoselective. Even monosubstituted, internally disubstituted, trisubstituted, tetrasubstituted and tetrasubstituted alkenes, as well as dialkenes, are oxidized to generate carbonyls or their methanol-protected form, dimethyl acetals. An unsaturated fatty acid derivative, for example, was cleaved into an aldehyde, which was then transformed *in situ* into the acetal. In addition, cyclopentene was ring-opened to produce diketone. The catalyst can be used to oxidize a variety of natural compounds, including pinene, nootkatone, vitamin K1 and others. The photocatalyst’s conceivable mechanism is as follows (Scheme 20): when blue lightimpinges on the Mn(II) catalyst **(I)**, it gets excited to **(II)**, which subsequently interacts with O_2_ to generate the Mn(III)-superoxo species **(III)**. The **(III)** removes hydrogen from methanol to produce intermediate **(IV)**, which then produces the Mn(IV)-oxo **(V)**, releasing formaldehyde (**34**) and water. In the meantime, the catalytically active **(V)** moves to the more stable bis–μ–O_2_–Mn_2_ complex **(VI)**. The **(V)** adds to the alkene **35** to form a new radical species **(VII)**, which is active towards O_2_, resulting in a six-membered metal-peroxo species **(VIII)**. The unstable **(VIII)** decomposes swiftly to give the product ketone **19** and the side product formaldehyde **34**, as well as regenerating **(V)** [48].

**Scheme 21.**
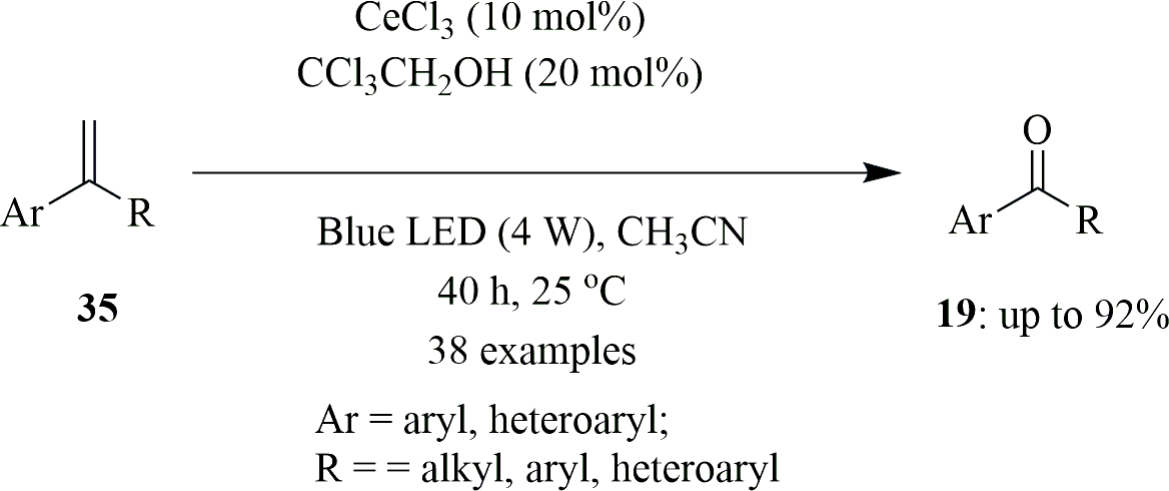
Oxidative cleavage of C=C double bonds for the synthesis of biaryl methanone via CeCl_3_ catalysis.

CeCl_3_ is another photocatalyst for the oxidative breaking of C–C double bonds using air as an oxidant to produce ketones (Scheme 21). This Ce-based photocatalytic reaction tolerates biaryl olefins **35** containing EDGs such as methyl and alkoxyl groups at the *ortho*-, *meta*- and *para*-positions of the phenyl ring, producing their corresponding substituted ketones **19** in good yields, just like the prior reaction. Furthermore, under normal conditions, substrates having EWGs such as F, Cl and nitro groups at any location of the phenyl ring are effective. Owing to the strong electron-withdrawing effect of the pyridine group, the catalyst has medium to good reactivity towards various styrenes but low reactivity towards pyridine-substituted olefins. Moreover, even if the catalyst loading is reduced to 0.5 mol%, the reaction delivers good results when the reaction duration is extended. This reaction takes place via an electron transfer pathway, in which the Ce^III^ species is photoexcited to its excited state, reducing O_2_ to a superoxide radical anion and generating a Ce^IV^ intermediate. The reaction of alcohol with the Ce^IV^ species and the subsequent photoinduced ligand-to-metal charge transfer produce highly electrophilic alkoxy radicals by releasing a proton. This radical removes one H atom from the olefin, resulting in an olefin radical, which then reacts with the proton to form a radical cation intermediate. The dioxetane intermediate is then formed by the [2 + 2] cycloaddition of an alkene radical cation with a superoxide radical anion, followed by the formation of the corresponding carbonyl product and by-product formaldehyde [49].

Under light irradiation, Hu *et al*. produced a photoelectrocatalyst for methanol oxidation in methanol and potassium hydroxide solution. Pt nanoparticles were dispersed on the surface of 2D perovskite La_2_Ti_2_O_7_ (LTO) nanosheets to make the catalyst. Both direct and indirect pathways are used in the photo-assisted methanol oxidation reaction, with formate and carbon monoxide as reactive intermediates and carbon dioxide as the oxidized result. The processes of oxidation in the first pathway are as follows:


$$(CH_{3}OH{)}_{solution}\to Pt\;-\;(CH_{3}OH{)}_{ads},$$

$$Pt\;-\;(CH_{3}OH{)}_{ads}+5OH^{-}\to Pt–HCOO_{ads}+4H_{2}O+5e^{-},$$

$$Pt\;-\;HCOO_{ads}\to Pt+CO_{2}+H^{+}+e^{-},$$

$$Pt\;-\;HCO_{ads}\to Pt–CO_{ads}+H^{+}+e^{-}$$

$$Pt+OH^{-}\to Pt–OH_{ads}+e^{-}$$

$$Pt\;-\;CO_{ads}+Pt\;-\;OH_{ads}\to CO_{2}+2Pt+H^{+}+e^{-}.$$

In the other pathway, light irradiation excites the LTO nanosheets, resulting in electron–hole pairs with electrons in the conduction band and holes in the valence band of the LTO. As illustrated in the stages below, the created holes (h) have significant oxidizing ability and can oxidize the adsorbed methanol molecules and intermediates [50]:


$$LTO+h\nu \to LTO+e^{-}+h^{+},$$

$$CH_{3}OH+6h^{+}+6OH^{-}\to CO_{2}+5H_{2}O.$$

$$Intermediates\;(CO_{ads})+OH^{•}\to CO_{2}+H^{+}+e^{-}.$$

We can oxidize benzene to create carbon dioxide in the same way that we can oxidize methanol by modifying the photocatalyst. The oxidation takes place in the presence of tungsten-doped MnO_2_ under xenon-lamp irradiation at temperatures ranging from 200 to 300°C. The introduction of W species into the MnO_2_ lattice promotes the photo-assisted reaction in two ways: firstly, it creates abundant oxygen vacancies (VO) on MnO_2_, which can serve as active sites for the chemisorption of O_2_, and secondly, the doped W atoms form strong covalent interactions with neighbouring O atoms via W–O bridging bonds, allowing O_2_ polarization and electron transfer. When exposed to light, photo-generated holes aid the adsorption of molecular benzene on O atoms at VO sites. By absorbing extra electrons from photoexcitation processes, the photoexcited electrons are easily trapped by the oxygen molecules, creating superoxide (^•^O^2−^) radicals. Simultaneously, h^+^ assists benzene ($C_{6}H_{6}^{+}$) adsorption and initial activation. The superoxide radicals can combine with molecular benzene to yield reactive benzene species ($C_{6}H_{6}^{*}$), which get oxidized to carbon dioxide by lattice oxygen at high temperatures, as shown in the steps below [51]:


$$O_{2}+e^{-}\to^{•}\;\;O^{2-},$$

$$C_{6}H_{6}+h^{+}\to C_{6}H_{6}^{+},$$

$$C_{6}H_{6}^{+}{+}^{•}\;O^{2-}\to C_{6}H_{6}^{*},$$

$$C_{6}H_{6}^{*}+lattice\;O\to CO_{2}+H_{2}O.$$

## 12. Silver-catalysed

**Scheme 22.**
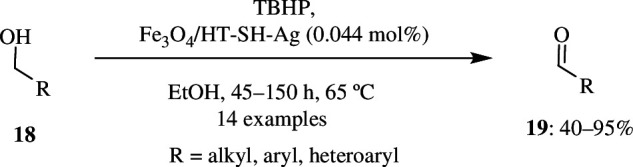
Oxidation of chlorobenzyl alcohol by *tert*-butyl hydroperoxide (TBHP) in the presence of Fe_3_O_4_/HT-SH-Ag catalyst.

Recently, there have been numerous efforts put forth to make all industrial organic reactive pathways eco-friendly. Hydrotalcite (HT) is considered to be a catalyst forerunner for *trans*-esterification and aldol condensation. It is also known that oxidation reactions in the presence of transition metals improve the efficiency. The oxidation of alcohols to corresponding carbonyl compounds is a very important reaction because these carbonyl compounds have numerous applications, mainly as precursors in several industries. Salimi *et al.* modified the catalyst HT to Fe_3_O_4_/HT-SH-Ag, and this was characterized by several instrumental analyses. The crystal structure of this catalyst was found using X-ray diffraction, and the size of the catalyst was estimated to be 27 nm using Debye–Scherrer’s equation. Using field emission scanning microscopy (FE-SEM) analysis, the catalyst was found to have irregular plate-like morphology. The catalytic activity was studied by taking chlorobenzyl alcohol (**18**) as the substrate followed by its oxidation to produce the corresponding aldehyde (**19**) (Scheme 22). Initially, the reaction was carried out in the absence of the catalyst, but in the presence of TBHP in ethanol as a green solvent at 65°C. However, even after 120 min, the required product was not formed. Later, the reaction was carried out in the presence of the catalyst, and it was found that maximum yield was obtained in a very short period of time. A total of 0.25 mmol of 4-chlorobenzyl alcohol **18** was oxidized by 0.25 mmol of TBHP in the presence of 0.044 mol% of the catalyst, Fe_3_O_4_/HT-SH-Ag and ethanol as green solvent, which resulted in the formation of 90% of product at 65°C when the reaction was carried out for 45 min. The oxidation of alcohol did not occur/resulted in a very much smaller percentage of product with oxidants like O_2_, H_2_O_2_, O_3_ or air. It was seen that aromatic alcohols with electron-releasing groups react faster than the ones with electron-donating groups. The catalyst was recyclable and was used for six continuous runs with negligible reduction in efficiency. The newly produced catalyst contained 1.70 mmol of Ag per 1 g of Fe_3_O_4_/HT-SH-Ag and 1.615 mmol of Ag per 1 g of Fe_3_O_4_/HT-SH-Ag after the sixth use. The recyclability was further confirmed by Fourier transfor infrared (FT-IR) analysis of the recovered catalyst, which showed a similar spectrum to the newly synthesized catalyst. The catalyst was easily separated out by an external magnet and was found to be stable against moisture and air [52].

## 13. Iron-catalysed

**Scheme 23.**
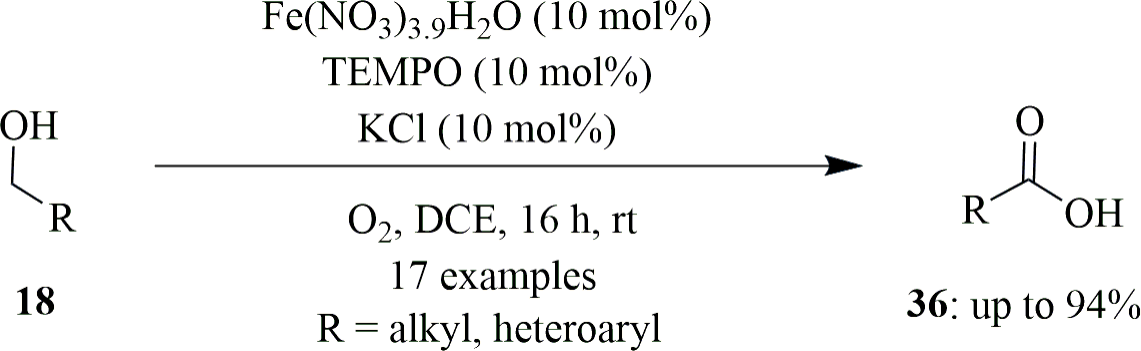
Direct oxidation of alcohol to acid using O_2_ as oxidant.

**Scheme 24.**
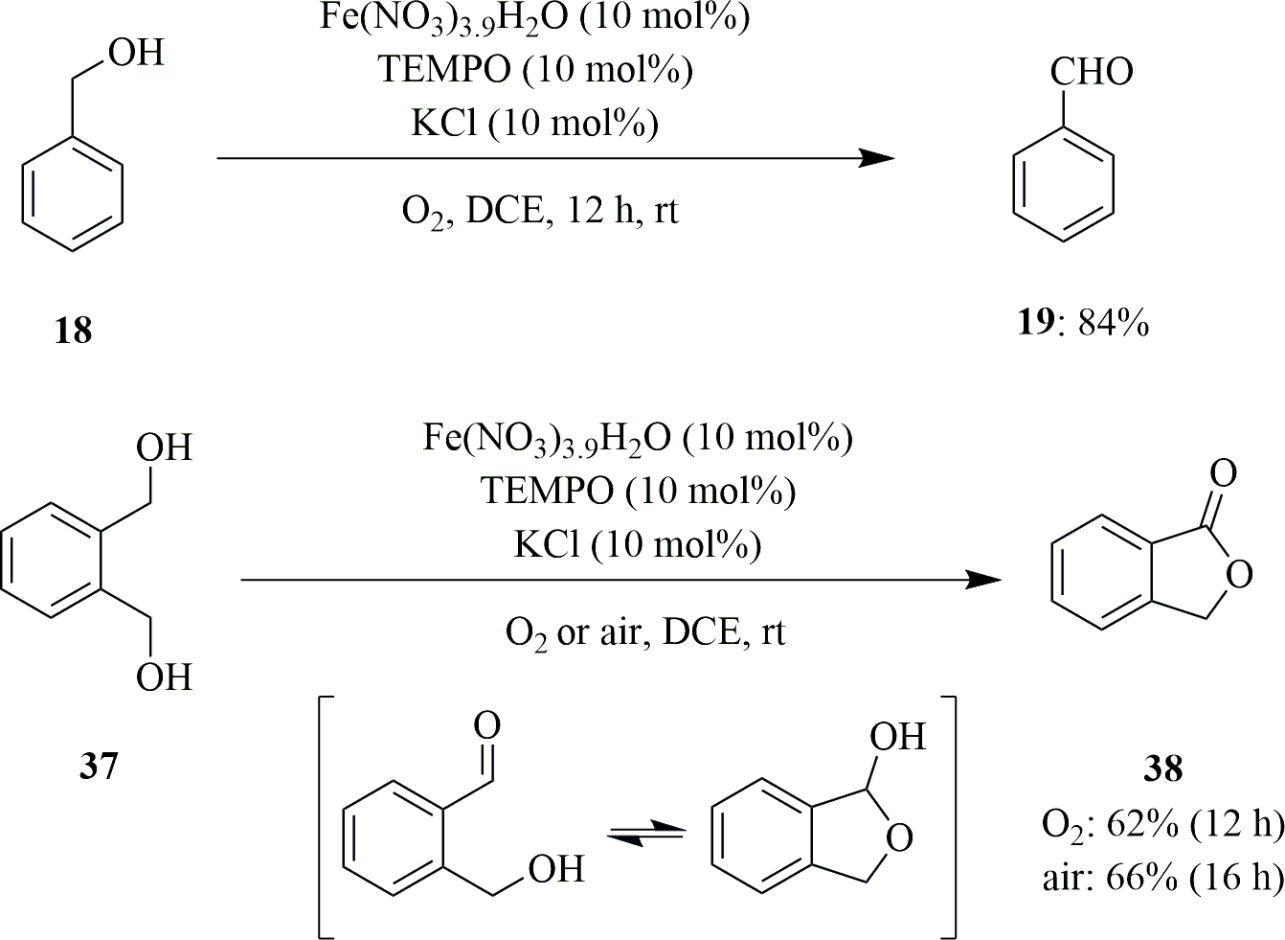
Oxidation of benzyl alcohol (**18**) and 1,2-benzenedimethanol (**37**).

Jiang *et al*. developed a method to produce carboxylic acid **36** by aerobic oxidation of alcohol **18** (Scheme 23). They used an iron catalyst and 2,2,6,6-tetramethylpiperidine-*N*-oxyl (TEMPO) with the clean, economic oxidant, O_2._ They isolated 95% palmitic acid by direct oxidation of cetyl alcohol in the presence of 10 mol% of Fe(NO_3_)_3_·9H_2_O, TEMPO and KCl with an oxygen gas-filled balloon in DCE for 16 h at room temperature (Scheme 23). The team screened various other nitrates and found that Co(NO_3_)_3_·6H_2_O, Ni(NO_3_)_3_·6H_2_O and Zn(NO_3_)_2_·6H_2_O were not effective. It was also concluded that Cu(NO_3_)_2_·3H_2_O catalysed the formation of 84% of the acid product and 16% of the aldehydic product after 48 h. This was also applied for oxidation of benzyl alcohols and 1,2-benzenedimethanol (**37**). Benzyl alcohol produced the respective aldehyde, whereas the 1,2-benzenedimethanol (**37**) produced lactone **38** through the formation and oxidation of hemiacetal (Scheme 24) [53].

**Scheme 25.**
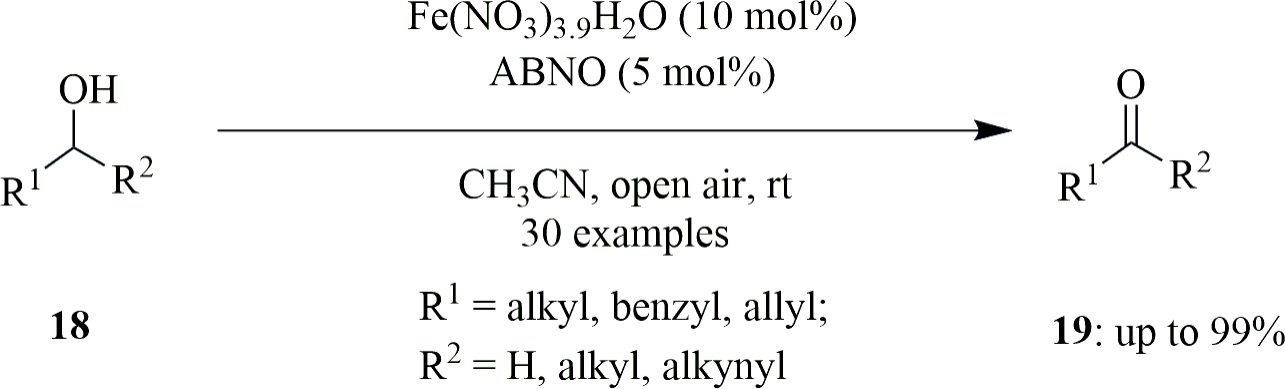
Aerobic oxidation of a broad range of primary and secondary alcohols to aldehydes and ketones.

Most of the Fe/TEMPO systems use halogenated hydrocarbon as solvent, which is a big disadvantage. In addition to this, they have very moderate selectivity towards aliphatic aldehydes owing to their potential to be over-oxidized to carboxylic acids. This may be due to lower catalytic activity of TEMPO and can be solved by using nitroxyl radical instead of TEMPO. These radicals are of good use for oxidation in the absence of Cu or transition metals and show high reactivity in contrast to TEMPO owing to less steric hindrance surrounding the *N*-oxyl radical and improved stability. Motivated by these results, Wang *et al.* reported the very first, highly selective and active Fe(NO_3_)_3_/ABNO (9-azabicyclo[3.3.1]nonane-*N*-oxyl) catalyst system for the aerobic oxidation of a very wide range of alcohols, including unactivated aliphatic, allylic and benzylic alcohols **18** to aldehydes and ketones **19** with ambient air as oxidant and acetonitrile as solvent at room temperature (Scheme 25). Oxidation of alcohols with various functional groups like alkyl halide, alkyl ethers, aryl and alkenes produced corresponding aldehydes in moderate yields with good selectivity. Similarly, secondary alcohols gave high yields of ketones [54].

**Scheme 26.**
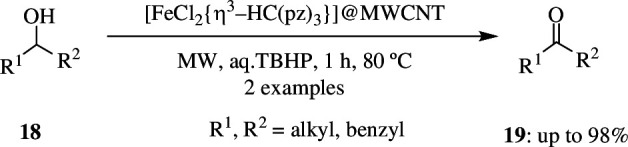
Oxidation of secondary alcohols to the corresponding ketones catalysed by C-MWCNT.

Naturally occurring iron-containing enzymes like cytochrome P-450 catalyse several reactions and oxidation of alcohol is one among them. Use of such naturally occurring compounds can mimic green procedures and hence is eco-friendly. Martins *et al.* made use of one such bio-inspired tris(pyrazol-1-yl)methane Fe(II) complex, FeCl_2_(η^3^-HC(pz)_3_) supported on multi-walled carbon nanotubes, which was oxidized with nitric acid followed by treatment with sodium hydroxide, to give the catalyst, and environmentally friendly TBHP was used as oxidant with MW irradiation as the source of energy [55]. Immobilization of the iron complex occurred by the formation of a covalent Fe–O bond. The complex-MWCNT, (C-MWCNT) system was tested by using it as a catalyst for oxidation of secondary alcohol **18** with aqueous 70% TBHP as oxidant. Alternatively, TEMPO was used as radical. After 1 h, the process resulted in the formation of respective ketone **19**. In the absence of the Fe(II) complex, the yields were very lower compared with over 98% yield produced in the presence of C-MWCNT (Scheme 26). One of the main advantages of the C-MWCNT is that it maintains catalytic efficiency even after several consecutive cycles of usage.

**Scheme 27.**
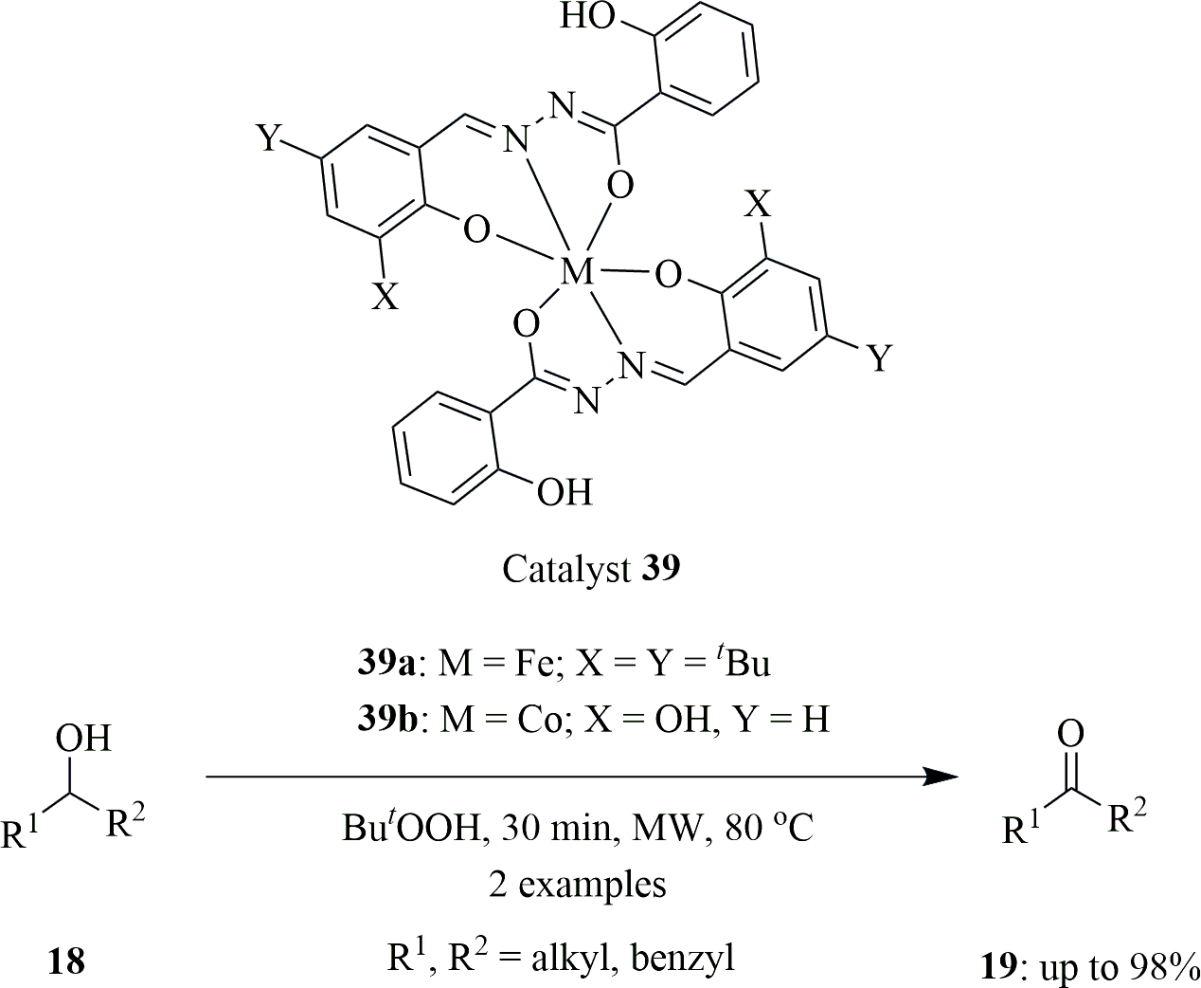
Solvent-free oxidation of secondary alcohols to their respective ketones.

The Schiff base derived by condensation of 2-hydroxybenzohydrazide with 3,5-di*tert*-butyl-2-hydroxybenzaldehyde, (H_2_L^1^), was utilized to synthesize Fe(III) complex [Fe(L^1^)(HL^1^)] **39**. The catalytic activity shown by the complex towards the oxidation of alcohols was utilized to carry out the process in an environmentally friendly manner (Scheme 27). The catalytic activity was supported by its study on 2.5 mmol of 1-phenylethanol **18**, which resulted in the formation of acetophenone **19** under mild oxidizing conditions using 5 mmol of TBHP as oxidant at 80–120°C with low power (5–15 W) MW irradiation in the absence of solvent.

The entire process depends on the temperature produced by the MW, which in turn is controlled by the MW power. It was observed that after 30 min, at 120°C, 92% of acetophenone **19** was obtained. When 2-pyrazinecarboxylic acid (Hpca), a co-catalyst, was added, there was an increase in the overall yield. In the presence of *n*(acid)/*n*[Fe(L^1^)(HL^1^)] = 5 under the same reaction conditions, 96% of acetophenone was obtained. The acid promotes the reaction by creating unsaturated metal centres [56].

**Scheme 28.**
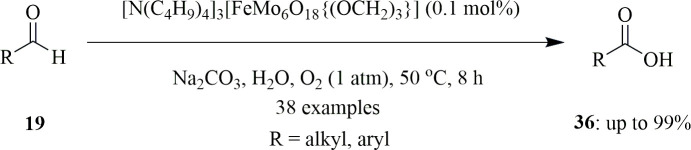
Iron(III)-catalysed aerobic oxidation of aldehydes.

Yu *et al.* used molecular oxygen as terminal oxidant owing to its abundance and greener characteristics. They utilized water as solvent for oxidation of aldehydes to corresponding acids. They employed 0.1 mol% of a single-sided triol-functionalized iron-centred Anderson polyoxometalate, [N(C_4_H_9_)_4_]_3_^−^[FeMo_6_O_18_(OH)_3_{(OCH_2_)_3_CNH_2_}] [Fe^III^Mo_6_], as catalyst for the oxidation of 1.5 mmol of benzaldehyde **19** with O_2_ at 1 atm in aqueous medium. The reaction was carried out at 50°C in the presence of additives, and the basicity of these additives had an influence on the percentage yield of benzoic acid (Scheme 28). It was found that when Na_2_CO_3_ having p*K*_b_ = 3.67 was used, 96% yield was obtained, and inclusion of additives with increased p*K*_b_ value decreased the percentage yield. Acidic additives like NH_4_Cl prevented the process of oxidation. These results suggested that the complex was influenced by the additives. The catalyst was recyclable and was used at least eight times without decrease in its performance. Excellent yields were produced with aromatic aldehydes, halogen-substituted aldehydes and several aliphatic aldehydes as well [57].

## 14. Copper-catalysed

**Scheme 29.**
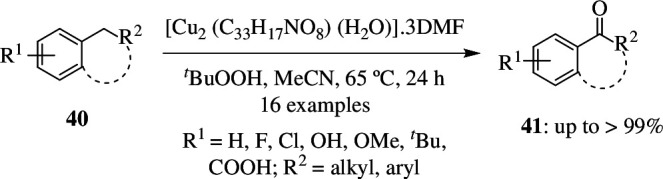
Catalytic oxidation of benzylic C–H compounds to the corresponding ketone using MFM-170 in the presence of ^*t*^BuOOH.

Most homogeneous catalysis utilizes complexes of transition metals and is hence costly and not environmentally friendly. But on the other hand, heterogeneous catalysis makes use of nanoparticles of precious metals that are supported on materials like silica, zeolites and titania, which are prone to deactivation due to aggregation and extensive leaching of the metal. But MOFs can hinder this and prevent the deactivation. These have single-site metal nodes that are joined by organic linkers and can be used as catalysts for various reactions. Kimberley *et al*. studied the oxidation process by making use of MFM-170, [Cu_2_(C_33_H_17_NO_8_) (H_2_O)]·3DMF in combination with external oxidant. Reaction was carried out with 0.25 mmol of indane **40** as substrate, 0.75 mmol of TBHP (3 equiv) as oxidant, 0.025 mmol of MFM-170 (10 mol%) as catalyst and 4 ml of acetonitrile at 65°C for 1 day. This resulted in the formation of 94% of 1-indanone **41** (Scheme 29). It was observed that negligible product was formed when oxidation took place in the presence of H_2_O_2_ or O_2_ as oxidant. In the presence of ^*t*^BuOOH as oxidant and without the MOF as catalyst, it was seen that a mixture of alcohol and ketone was formed. This establishes the importance of MFM-170 as a selective activating factor for ^*t*^BuOOH. The catalytic activity of other homogeneous catalysts based on simple salts like CuCl_2_, Cu(NO_3_)_2_ and Cu(OAc)_2_ was tested on the same substrate, and these yielded 34, 58 and 63% of 1-indanone, respectively. This highlights the importance of immobilizing Cu(II) ions into the paddlewheel moiety of the MOF, which in turn results in increasing the catalytic activity towards benzylic oxidation. MFM-170 shows very high stability and can be reused for several cycles without much decrease in its catalytic activity [58].

**Scheme 30.**
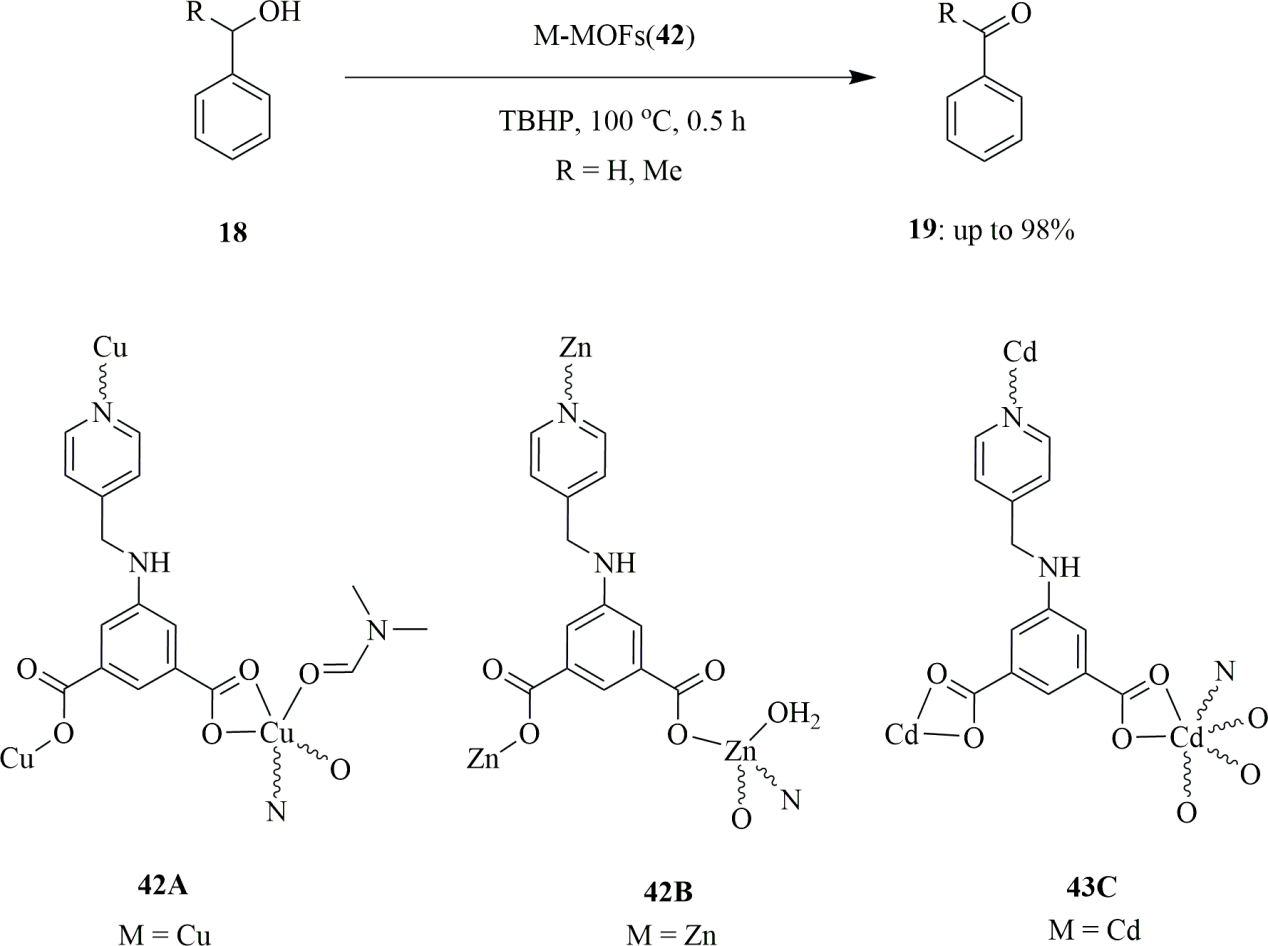
Solvent-free microwave-assisted oxidation of alcohol **18**.

Another group used 5-{(pyridin-4-ylmethyl)amino}isophthalic acid (H_2_L^1^) as an organic linker to form MOF with different metals like copper(II) (**42A**), zinc(II) (**42B**) and cadmium(II) (**42C**). Karmakar *et al*. selected this ligand because one can introduce an amine functionality that will in turn provide an additional hydrogen binding site. It is also possible to obtain various architectures with this ligand owing to the ability of the pyridyl arm to change its conformations. Additionally, by changing the pH of the medium, the rate of deprotonation of the carboxylic group in the ligand can be tuned easily. It was found that the catalytic activity of the Cu(II)-based MOF was the highest compared with the other two. Reacting copper(II) nitrate with the organic ligand **42A**, H_2_L1, in the presence of dimethyl formamide (DMF) and NH_4_OH resulted in the formation of [{Cu(L1)(DMF)}.DMF.H_2_O]_*n*_ (**42A**). The catalytic activity was tested with 5 μmol of catalyst on MW-assisted oxidation of primary and secondary alcohols **18**, i.e. benzyl alcohol or 1-phenylethanol to aldehyde or ketone **19** as benzaldehyde and acetophenone, respectively (Scheme 30). Two equivalents of aq. 70% TBHP was used as oxidant with 0.5 h of MW irradiation at 100°C. Gas chromatography was taken in the presence and absence of catalyst. It was found that no considerable amount of product was formed without catalyst. The catalyst was found to retain its catalytic activity for three consecutive cycles while maintaining its selectivity [59].

**Scheme 31.**
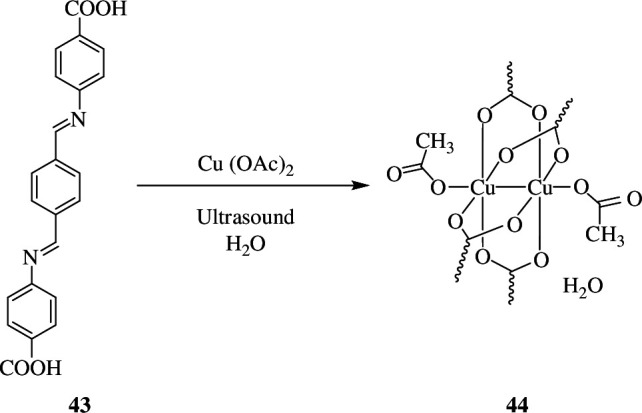
Synthesis of UoB-1 nanostructures **44** from Schiff base **43** as organic linker.

Aryanejad *et al*. synthesized a new Cu(II)-based nano-MOF, (UoB-1) **44**, with Schiff base 4,4'-[benzene-1,4-diylbis(methylylidenenitrilo)] (**43**) as the linker (Scheme 31).

**Scheme 32.**
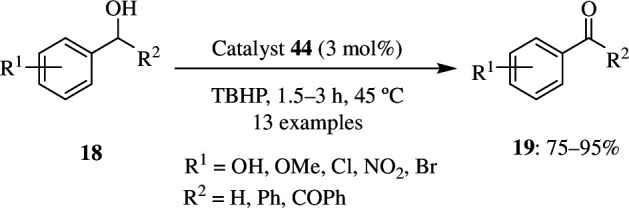
Oxidation of benzylic alcohols in presence of UoB-1 by TBHP.

Its catalytic activity in oxidation reaction was then tested by using 1 mmol of benzyl alcohol **18** as substrate along with 2 mmol of TBHP as oxidant and 3 mol% UoB-1 nanostructures **44** as catalyst at 45°C for 2 h in the absence of any solvent (Scheme 32). This resulted in the formation of 95% benzaldehyde **19**. This was also carried out with various other aryl alcoholic substrates that had substituted EDGs and EWGs. The reaction rate was faster when EDGs were substituted and the *para* position was found to be more active than the *ortho* position. When the temperature was increased beyond 45°C, i.e. to 65 and 85°C, over-oxidation occurred, which resulted in the formation of acids corresponding to the substrate used. In the absence of the MOF as catalyst, only moderate yields of aldehyde or ketones were formed. This nanosized UoB-1 could be recycled and reused for six cycles without any decrease in its catalytic activity [60].

**Scheme 33.**
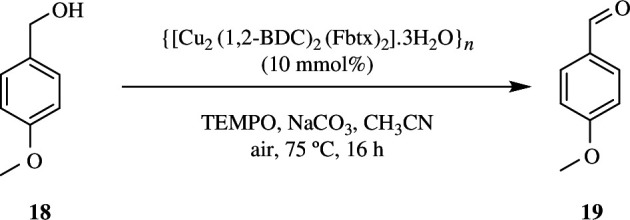
Aerobic oxidation of alcohol by using Cu-FMOF.

There have been several advancements in the field of heterogeneous catalysis. However, use of air or O_2_ as green oxidant for alcoholic oxidations is still an issue. Chen *et al*. have synthesized a Cu-based MOF, {[Cu_2_(1,2-bdc)_2_(Fbtx)_2_]·3H_2_O}_*n*_, (Cu-FMOF), where 1,2-bdc is 1,2-benzenedicarboxylate and Fbtx is 1,4-bis(1,2,4-triazol-1-ylmethyl)-2,3,5,6-tetrafluorobenzene. Oxidation of 12 µl, 0.1 mmol of 4-methoxybenzyl alcohol was done utilizing 11.3 mg, 10 mmol% of Cu-FMOF as catalyst with 7.8 mg, 0.05 mmol of TEMPO and 10.6 mg, 0.1 mmol Na_2_CO_3_ in 1 ml of air saturated with acetonitrile. The solution was magnetically stirred for 16 h at 75°C under air atmosphere in a round-bottom flask. This led to complete conversion of 4-methoxybenzyl alcohol **18** into 4-methoxybenzylaldehyde **19** (Scheme 33). The high activity of the catalyst maybe due to the presence of five-coordinated Cu(II) ions and mixed linkers. Oxidation of secondary alcohols produced lesser yields of ketones. Temperature-variable powder X-ray diffraction analyses confirmed that Cu-FMOF was stable up to 200°C. Just like the previously mentioned MOF catalysts, this catalyst could also be recycled and reused for five consecutive cycles [61].

**Scheme 34.**
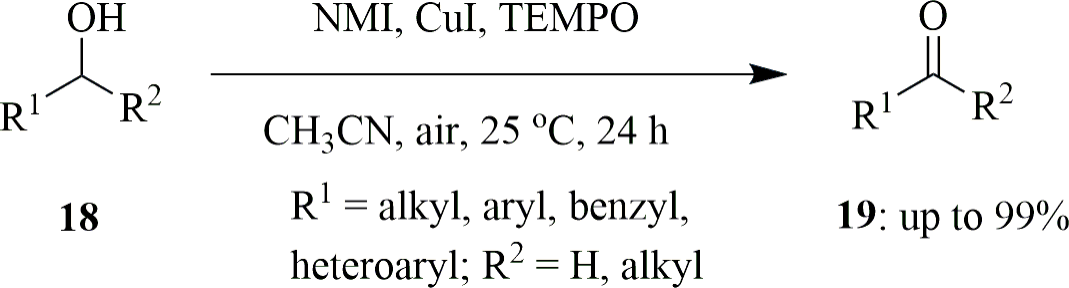
Aerobic oxidation of alcohols **18** to respective aldehydes **19**.

Aerobic oxidation of alcohols to their corresponding carbonyl groups in ambient conditions is an important aspect. It has been seen that Cu(I)/nitroxyl catalyst systems have high efficiency in oxidizing a variety of alcohols. Out of many combinations, 2,2′-bipyridine (bpy) along with excess of *N*-methylimidazole (NMI) has proved to be the most common system. Usually, NMI is used as organic base. But Liu *et al*. investigated the role of NMI as catalyst for reactions using CuI and [Cu(CH_3_CN)_4_]PF_6_ as the Cu(I) source in the Cu(I)/NMI/TEMPO system. The Cu centre will be bound by two NMI ligands or one iodide and one NMI ligand along with two labile ligands from the solvent, which is said to be the main components of its catalytic activity. As the electron density on the metal centre increases, the catalytic nature also increases owing to the activation of O_2_ by electron transfer. The catalytic system was tested by oxidation of alcohols **18** as 1-octanol to respective aldehyde **19** at room temperature in acetonitrile with air as the oxidant. In total, 89% of the product was formed after 24 h when the Cu(I)/NMI/TEMPO ratio was 1 : 2 : 1 (Scheme 34). The reaction was said to be solvent-dependent, and so find the most suitable solvent for the reaction, it was carried out in the presence of several solvents: DCM, DMSO, DMF, THF and CH_3_CN. It was concluded that acetonitrile (CH_3_CN) was the most suitable solvent as it gave 89% of the product as compared with 79% for DCM, 73% for DMSO and so on. Most of the common oxidation reactions are carried out in the presence of base, but in this system, use of such bases deteriorated the oxidation process and resulted in lower yield, which might be due to the fact that bases may decrease the available copper species by forming their corresponding hydroxides. This system was found to be useful for oxidation of various benzylic-type alcohols with varying substrates, be it EDG or EWG, and aliphatic primary alcohols. Apart from this, it showed some oxidative activity towards 2-phenylethanol but almost nil activity against any other secondary alcohols [62].

**Scheme 35.**
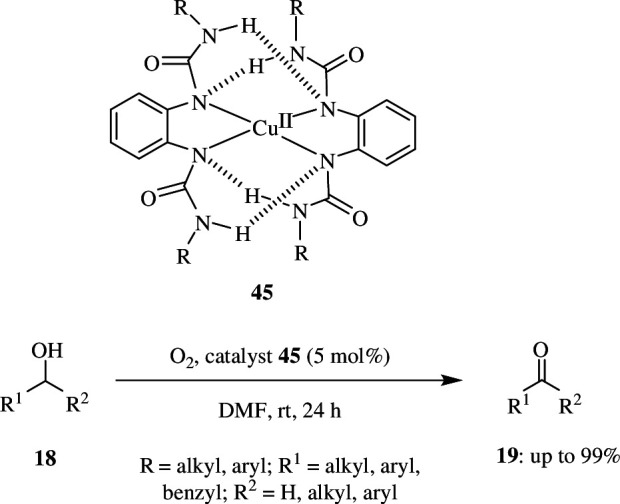
Dehydrogenation of alcohols catalysed by copper complexes.

Metal complexes with ligands that are redox-active have a wide a range of applications, including acting as catalysts for various reactions. Rajabimoghadam *et al*. synthesized several bidentate ligands such as diamido-uranyl with varying alkyl groups. Using these ligands, copper complexes were formed. These complexes were able to stabilize several oxidation states via oxidation of the central metal, which resulted in galactose-oxidase-like oxidation of alcohols. This was tested by carrying out oxidation of benzyl alcohol using O_2_ as oxidant giving benzaldehyde as the product. The complexes that showed higher oxidation states at lower potential (^*t*^BuPhCu^II^) gave excellent yields, nearly 90–96% as compared with the 0–30% yield given by the complexes that were oxidized at higher potential. Oxidation was also carried out using other substrates with ^*t*^BuPhCu^II^ as catalyst. Very good oxidation yields were obtained when the substrates were dibenzyl methanol, benzoin and cinnamyl alcohol. These had weak C–H bonds with acidic O–H. In contrast, when cyclohexanol, with strong C–H, and 1-phenyl ethanol, having basic O–H, gave very poor yields or were not oxidized at all (Scheme 35) [63].

**Scheme 36.**
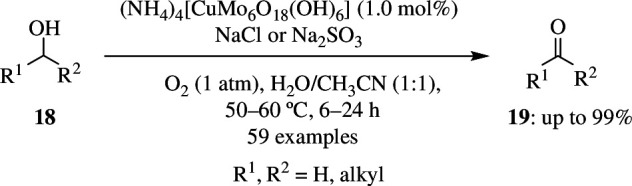
Cu-catalysed oxidation of alcohols.

Most oxidation reactions are catalysed by organometals and employ nitroxyl radicals as co-catalysts. The majority of these require a large number of organic compounds, which makes the oxidation a very expensive process, especially when carried out at the industrial level. Wei *et al*. reported that Anderson-type polyoxometalates could be used as an inorganic ligand, supporting metals as catalysts for oxidation reactions. The catalyst C1, (NH_4_)_4_[CuMo_6_O_18_(OH)_6_], was used as an alternative for the greener conversion of alcohols to corresponding carbonyls with O_2_ as sole oxidant in a mixture of solvents, acetonitrile and water. The study was done with 1.0 mmol alcohol **18** as piperonyl alcohol, using 1.0 mol% of catalyst and O_2_ at 1 atm as oxidant, with water as solvent and acetonitrile as co-solvent (v/v = 1 : 1) at 60°C (Scheme 36). This resulted in the production of 90% of oxidized product **19** within 12 h. The efficiency of the reaction was not improved by increasing the proportion of water but was influenced by additives like salts bearing Cl^−^ ions, which gave excellent yields of >99%. The reaction conditions did not give any over-oxidized product, and it was found that decreasing the catalyst loading from 1 mol% to 0.5 or 0.1 mol% decreased the aldehydic product. Good yield was obtained when the reaction was carried out at pH in the range of 5–9. Going beyond this resulted in decomposition of catalyst because of its dependence on pH for its structure. The conversion rate was less when the medium was too acidic or basic. Several other substrates, like alcohols having a C=C double bond or terminal C≡C triple bond, single-ring alcohols and polycyclic alcohols that are sterically hindered, can be oxidized using a similar approach. However, linear alcohols like octan-1-ol were over-oxidized to carboxylic acids under these conditions. This catalyst is highly stable and can be used without any degradation for a minimum of six times [64].

**Scheme 37.**
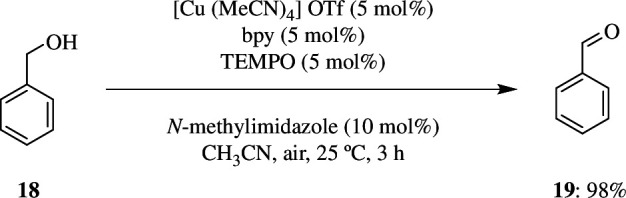
Cu-catalysed Stahl oxidation.

[Cu(MeCN)_4_]OTf, which was prepared by an electrochemical method, was found to be useful in aerobic oxidation of alcohols. A stock solution of 0.15 mmol of complex with 1.5 ml of MeCN was added equally to another stock solution present in two separate vials each containing 3.0 mmol of benzyl alcohol **18**, 0.15 mmol bipyridine, 0.15 mmol TEMPO, 0.30 mmol of NMI and 1.5 ml MeCN. The mixture was stirred magnetically for 3 h at 25°C. In each of the vials, the oxidation product **21** was found to be 97 and 98% (Scheme 37) [65].

**Scheme 38.**
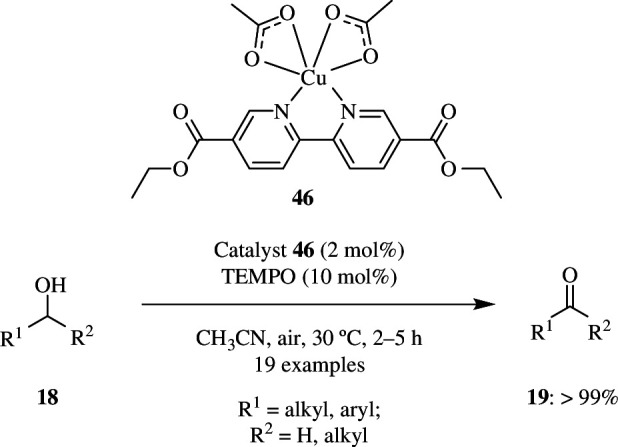
Cu-catalysed oxidation of alcohols.

There are several metals that can be utilized as catalysts for oxidation reactions. But they either need external alkali and elevated temperature or are costly. However, copper-based catalysts are advantageous because they were found to overcome such drawbacks. Cu(BPYDCDE)(OAc)_2_(**46**), a complex, was synthesized from the ligand [2,2']-bipyridinyl-5,5'-dicarboxylic acid diethyl ester (BPYDCDE) and a metal precursor Cu(OAc)_2_. This was found to be an effective catalyst for oxidation of alcohols into their corresponding aldehydes or ketones at ambient temperature without any external base and using air as oxidant (Scheme 38). The potential of the catalyst was observed by comparing the yield of benzaldehyde **19** obtained by oxidation. When the Cu catalyst was used, the product yield was >99% in 2 h, whereas when Ni, Co and Zn were used instead of the copper complex, almost no benzaldehyde **19** was obtained. It was also found that the catalyst had high selectivity towards benzaldehyde, and hence no benzoic acid was formed. The catalytic activity was not lost after up to five times of reusage.

In the studies done, it was observed that the product yield was dependent on the alkalinity of the counterion of the complex and the ligand structure. Here, the p*K*_a_ of HOAc is 4.75, and hence the yield was high, but when the counterions were changed and the p*K*_a_ value decreased, the yield decreased drastically. As mentioned earlier, oxidation reaction using this catalyst does not need an external base because the OAc^−^ in the complex in itself acts as base, and the catalyst proved to be more efficient even when lower amounts were involved. This catalyst was also effective for oxidizing secondary alcohols and benzyl alcohols substituted with EWDs and EDGs [66].

**Scheme 39.**
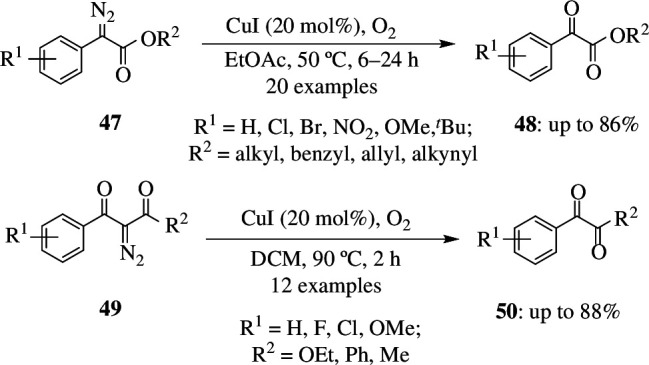
Cu(I)-catalysed aerobic oxidation of α-diazoesters **47** and **49**.

α-Ketoesters have various advantages, including their importance in several biologically active compounds, their usefulness as intermediates in transformations of functional groups, and so on. They can be obtained by oxidative dedinitrogenation of α-diazoesters. The oxidation of diazoesters to ketoesters has limitations owing to low product yield. Xu *et al.* developed an efficient catalytic oxidation of α-diazoesters to α-ketoesters involving a copper carbene intermediate. Ethyl phenyldiazoacetate **47** was oxidized to **48** in the presence of EtOAc as solvent at 50°C for 7 h with CuI as catalyst and oxygen gas-filled balloon as oxidant. When the reaction was carried out at room temperature, only 57% of **48** was formed, while increasing the temperature to 50°C resulted in unwanted *E-* and *Z*-alkenes, which were reduced to trace amounts by using optimum conditions to carry out the reaction (Scheme 39). It was also found that EWGs and EDGs when used as substituents on the phenyl ring tolerated the reaction and gave moderate yields [67].

## 15. Photocatalysis using Ti

**Scheme 40.**
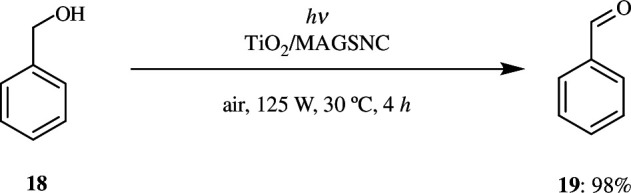
Selective oxidation of benzyl alcohol **18** to benzaldehyde **19** by magnetically separable TiO_2_/maghaemite–silica photo-active nanocomposite.

The popular chemical oxidation techniques have several drawbacks with respect to selectivity and availability. To overcome this, research teams have come up with methods that are more cost-effective, clean and efficient. Photocatalytic oxidation is one such way that can be implemented to produce carbonyl compounds, particularly aldehydes, by oxidizing alcohols. The most widely used photocatalyst is TiO_2_ owing to its photo-generated holes, which are considered to have high oxidizing power, its inertness, and so on. It is doped with noble metals like Pt, Ag and Au in order to enhance its activity as a catalyst by improving its tendency to absorb light and delay the process of recombination between the electron–hole pairs. Colmenares *et al*. prepared TiO_2_ carried on a magnetically separable support, maghaemite–silica nanocomposite (MAGSNC), by a wet impregnation method along with ultrasonic irradiation. This showed good catalytic activity, selective for oxidation of benzyl alcohol to its corresponding aldehyde, in both organic and aqueous media. Photolysis of benzyl alcohol in the absence of catalyst gave <5% of oxidative product after 4 h (Scheme 40). Similarly, using MAGSNC support as catalyst gave very low yield, which indicated the fact that γ-Fe_2_O_3_ does not have any significant use during the photooxidation process owing to the fact that it promotes recombination of electron–hole pairs, which in turn results in the formation of inactive materials. But TiO_2_/MAGSNC together as catalyst in acetonitrile medium improved the product yield and resulted in the formation of up to 48% conversion to product with 98% selectivity after 2 h of illumination. When the solvent was aqueous medium, the yield was low, and hence it was confirmed that yield was dependent on the solvent used. Certain solvents like acetonitrile increase the yield owing to a shielding effect. One of the major advantages of the catalyst is that it is magnetically separable and can be reused a number of times [68].

**Scheme 41.**
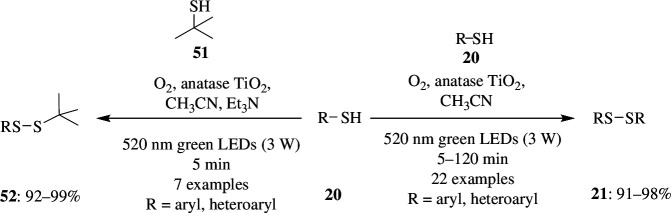
Visible-light photocatalytic selective aerobic oxidation of thiols to disulfides with O_2_ on anatase (TiO_2_).

Disulfide bonds play a major role in biological systems and in various industries as dopants in polymeric and rubber materials. One of the major strategies for their synthesis is the oxidation of thiols in the presence of stochiometric amounts of oxidants like H_2_O_2_, halogens and sulfoxide, which generate a lot of waste. To overcome this, one can use O_2_ as oxidant, and in order to increase the activity and selectivity, thermally activated pathways are said to be more efficient. But because biologically active compounds are sensitive to high temperature, this method was less preferred, and a visible-light photocatalytic pathway was found to be advantageous when it came to carrying out a reaction at room temperature and in a green way, especially in the presence of a heterogeneous catalytic system. Xu *et al*. established a method that utilizes green LEDs as the light source to carry out the reaction, with O_2_ on TiO_2_ as electron and proton acceptor instead of being incorporated into substrates. This transformation was effective in selective oxidation of thiols bearing EWGs (Scheme 41). This was tested by using 4-methylbenzenethiol **20** as the substrate, and TiO_2_ (ST-01) as the photocatalyst, in the presence of CH_3_CN as solvent. Under 520 nm green LED irradiation, 92% of the product **22** was obtained with excellent selectivity. The yield decreased to 74% when CH_3_OH replaced CH_3_CN as solvent, which might be due to its interaction with the substrate/TiO_2_ [69].

**Scheme 42.**
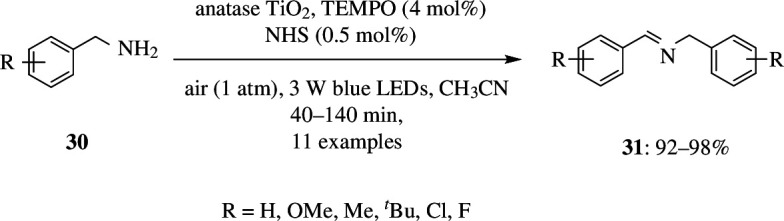
Visible-light-driven selective aerobic oxidation of benzylamine by cooperative TiO_2_ photocatalysis with TEMPO and NHS.

Recently, amines, particularly benzylamines, were assembled on TiO_2_, which led to their oxidation when illuminated with light of wavelength greater than 420 nm. In the visible-light catalysis, the efficiency of this self-assembly was very low, and it was found to be unstable owing to accumulation of charge on the TiO_2_ and very much lower electron transfer efficiency. Lyu *et al*. introduced TEMPO and N-hydroxysuccinimide (NHS) as redox mediators into the blue light-mediated self-assembled system. These accelerated the formation of oxidation products, and the catalyst stability was increased such that the TiO_2_ was found to be recyclable up to five times without loss of activity. The catalytic activity was proved by addition of TiO_2_ and benzylamine to acetonitrile and exposing this mixture to a blue LED of wavelength 460 nm (Scheme 42). Lyu *et al*. found that this led to selective oxidation of benzylamines to imines. Several *para*-substituted benzylamines also showed such reaction, wherein TEMPO and NHS acted as good co-catalysts. Additional experiments were carried out to find the role of these co-catalysts, which led to the conclusion that these did not have the ability to absorb visible light and hence could not be used in the absence of TiO_2_. Apart from this, the authors also found that the conversion increased as the amount of TiO_2_ was increased and that increasing the intensity of visible light improved the product yield [70].

## 16. Co-catalysed

**Scheme 43.**
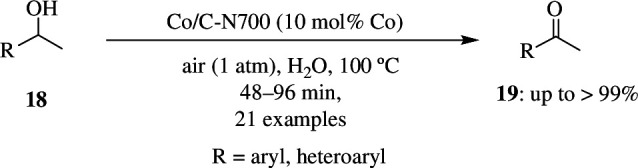
Oxidation of 1-phenylethanol **18** to acetophenone **19** by MOF-derived Co catalyst.

Several noble-metal-based catalysts are currently being used for oxidation reactions. Even though they give good yields, they are costly, and hence there is a need to shift to non-noble metals for catalysing reactions. But these require additional bases and explosive oxidizing agents to increase their efficiency and activity. Bai *et al.* have synthesized a Co-based complex that plays a major role as reusable catalyst in the oxidation of alcohols to carbonyl compounds in the presence of air as oxidant without the use of external base. The 10 mol% Co/C–N material prepared at 700°C was found to show the best catalytic activity for oxidation of 1-phenylethanol **18** when water was used as solvent (Scheme 43). This reaction was carried out using air as oxidant at 111°C for 48 h. In the absence of the catalyst, the reaction did not proceed. It was also seen that a much lower yield was obtained when activated carbon alone was used. When Co/C–N700 was used as catalyst, only 34% yield was seen. This shows the importance of N-doping in the activity of the material. The increase in activity when this catalyst is used is mainly due to the synergic interaction between C–N composite and Co [71].

Oxidations of several other substrates were also tested. Secondary benzylic alcohols having EDGs and EWGs gave very good yields of the corresponding carbonyl compounds within 48 h. Similarly, sterically hindered diphenylmethanol and other primary alcohols were also oxidized to ketones with good yield. Many other alcohols were also oxidized, even though they gave lower yields; it was seen that prolonging the reaction time increased the product yield.

**Scheme 44.**
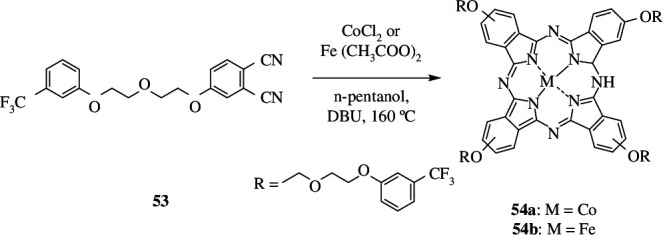
Synthetic route of phthalocyanine compounds **54**.

**Scheme 45.**
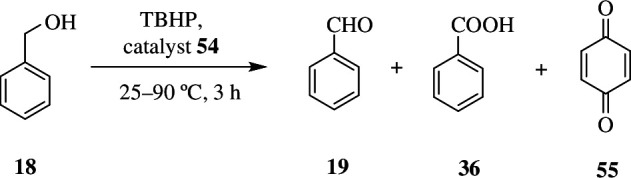
Oxidation of benzyl alcohol **18** by an oxidant in the presence of catalysts **54a** and **54b** (see scheme 44).

Aktaş *et al* synthesized Co(II) and Fe(II) phthalocyanines **54** (Scheme 44), whose catalytic activity for oxidation reaction was tested and compared by using benzyl alcohol **18** as substrate. Oxidation led to several minor products like benzoquinone **55** and benzoic acid **36**, and benzaldehyde **19** as major product. Benzyl benzoate was also produced in trace amounts. The reaction was carried out at 50°C for 180 min; this gave 98 and 90% of benzaldehyde with the Co(II) and Fe(II) complex, respectively (Scheme 45).

It was seen that product yield was decreased when substrate/catalyst ratio was increased, which was due to a smaller number of available catalytic sites. The ratio 600/1 was found to be optimum for good yield. Apart from this, even oxidants played a major role, wherein usage of H_2_O_2_ or *m*-CPBA degraded the complex, resulting in low conversion. But when TBHP was used as source of oxygen, good yields were produced. The reaction depended on the TBHP : benzyl alcohol ratio. The product yield was less when the ratio was increased in the presence of both the complexes. As the temperature was increased, starting from 25°C, to 90°C, there was an increase in the benzaldehyde **19** formed. The maximum yield was obtained at 50°C, and beyond 90^o^C the selectivity dropped owing to the complete conversion of benzyl alcohol. Of the two catalysts, Co(II) complex **54a** was found to be more effective than Fe(II) **54b**, owing to high d-electron density [72].

**Scheme 46.**
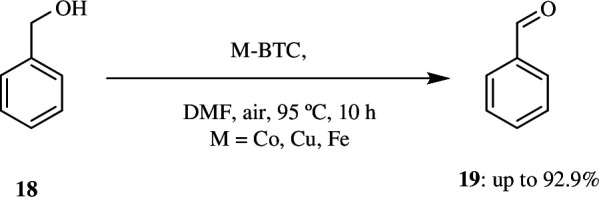
Oxidation of benzyl alcohol **18** by M-BTC catalyst.

Peng *et al*. developed new catalysts based on 1,3,5-benzenetricarboxylic acid (BTC) as ligand with cobalt, copper and iron (M-BTC) as central metals and tested their utility as catalyst for oxidative reactions. Here again, benzyl alcohol **18** was used as substrate for carrying out the reaction (Scheme 46). Dependence of the catalytic nature on temperature, solvent, time, etc., was also checked. In the absence of catalyst, even after 10 h, the conversion was only 3% when air was used as oxidant. When the reaction was carried out at 95°C for 10 h in the presence of 10 mg of the catalyst, 92.9% was the conversion and 97.1% was the benzaldehyde **19** selectivity. Out of the three, the cobalt complex was found to show better catalytic activity than Cu and Fe. Several solvents were tested, and finally, it was concluded that DMF was the most suitable solvent because it coordinated with the Co(II) centre, thereby promoting the activation of O_2_. The reusability of Co-BTC was tested by using the catalyst for the next consecutive reactions. It was seen that the yields reduced to 90 and 88% for the second and third cycles [73].

**Table 1.** A brief summary of the reactions.

	reactions	catalysts	references
1	oxidation of alkanes to alcohol 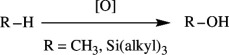	Ni, Ru	[20,24]
2	oxidation of alkanes to ketone 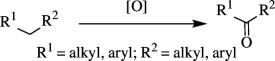	Mn, Cu	[31,59]
3	oxidation of alkene to ketone 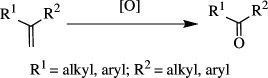	Ce, Mu	[49,50]
4	oxidation of alcohols 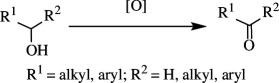	Mn, Fe, Ir, V, Cr, Co, Cu, Ti, photocatalytic	[28,34,45–47,53–56,60–67,69,72,73]
5	oxidation of carbonyl groups 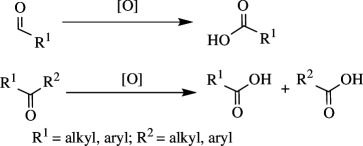	Ru, Fe, Co	[27,54,58,73]
6	oxidation of sulfur 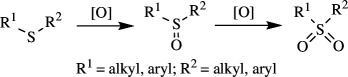	Pd, Cr	[35,42]
7	oxidation of thiols 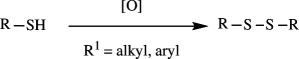	Rh, Cr, Pd, Ti	[32,35,42,70]
8	oxidation of aniline 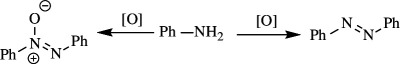	Mo, photocatalystic	[37,47]
9	oxidation of benzylamine 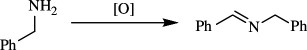	photocatalytic, Ti	[47,71]
10	oxidation of α-diazoesters 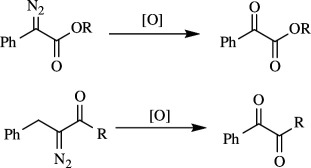	Cu	[68]
11	oxidative cyclization of diols 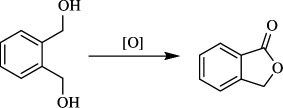	Fe	[54]
12	Oxidative trifluoroacetoxylation 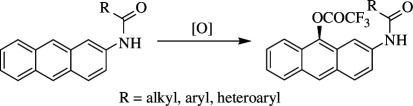	Rh	[33]

## 17. Conclusion

In this review article, we highlight the recent developments in several metal-based catalysts for oxidation reactions that play a crucial role in the chemical industry and are highly demanded owing to their role in essential functional group transformations. Major breakthroughs in the area of catalysis have been achieved with utilization of nanomaterials and MOFs leading to superior catalytic properties.

## Data Availability

This article has no additional data.
